# Fingerprinting heterocellular β-adrenoceptor functional expression in the brain using agonist activity profiles

**DOI:** 10.3389/fmolb.2023.1214102

**Published:** 2023-08-17

**Authors:** Rachel A. Matt, Frederick G. Westhorpe, Rosemary F. Romuar, Payal Rana, Joel R. Gever, Anthony P. Ford

**Affiliations:** Department of Pharmacology, CuraSen Therapeutics, San Carlos, CA, United States

**Keywords:** adrenergic, noradrenergic, GPCR, biased agonism, agonist, desensitization, receptor, expression

## Abstract

Noradrenergic projections from the brainstem locus coeruleus drive arousal, attentiveness, mood, and memory, but specific adrenoceptor (AR) function across the varied brain cell types has not been extensively characterized, especially with agonists. This study reports a pharmacological analysis of brain AR function, offering insights for innovative therapeutic interventions that might serve to compensate for locus coeruleus decline, known to develop in the earliest phases of neurodegenerative diseases. First, β-AR agonist activities were measured in recombinant cell systems and compared with those of isoprenaline to generate Δlog(E_max_/EC_50_) values, system-independent metrics of agonist activity, that, in turn, provide receptor subtype fingerprints. These fingerprints were then used to assess receptor subtype expression across human brain cell systems and compared with Δlog(E_max_/EC_50_) values arising from β-arrestin activation or measurements of cAMP response desensitization to assess the possibility of ligand bias among β-AR agonists. Agonist activity profiles were confirmed to be system-independent and, in particular, revealed β_2_-AR functional expression across several human brain cell types. Broad β_2_-AR function observed is consistent with noradrenergic tone arising from the locus coeruleus exerting heterocellular neuroexcitatory and homeostatic influence. Notably, Δlog(E_max_/EC_50_) measurements suggest that tested β-AR agonists do not show ligand bias as it pertains to homologous receptor desensitization in the system examined. Δlog(E_max_/EC_50_) agonist fingerprinting is a powerful means of assessing receptor subtype expression regardless of receptor expression levels or assay readout, and the method may be applicable to future use for novel ligands and tissues expressing any receptor with available reference agonists.

## 1 Introduction

The β-adrenergic receptor (β-AR) family comprises three established targets of successful therapeutics for patients with respiratory, cardiovascular, and urological disorders (Wachter and Gilbert, 2012; Camoretti-Mercado and Lockey, 2019). Historically, β-ARs have not been targeted for central nervous system-acting therapeutics, despite implications of early decline of noradrenergic tone in neurodegenerative disease (Leanza et al., 2018; Magistrelli and Comi, 2019). Brain β-ARs are activated by noradrenaline released principally from diffuse neuronal projections arising from the pontine locus coeruleus (LC) (Brunnström et al., 2011; Schwarz and Luo, 2015). The LC is especially vulnerable to neurodegenerative processes. It is the first brain region tau pathology of Alzheimer’s disease emerges (Braak et al., 2011) and one of the first to show alpha-synuclein/Lewy body pathology; subsequent loss of noradrenergic neurons is progressive and correlated with decline in cognitive function (Mather and Harley, 2016; Matchett et al., 2021). Noradrenergic tone is essential for wakefulness, arousal, regulation of sleep and mood, memory, and impulsive behaviors (Berridge and Waterhouse, 2003; Aston-Jones and Cohen, 2005; Bari and Robbins, 2013; Mather et al., 2016; Roozendaal and Hermans, 2017; Hayat et al., 2020). As excitatory targets of noradrenergic neurotransmission, β-ARs are known to have important neuronal (Mather et al., 2016) and non-neuronal functions, including astrocytic maintenance of nutrition and metabolism (Hertz et al., 2010; Dienel and Cruz, 2016), modulation of the neurovascular unit (NVU) and maintenance of cerebral perfusion (Toussay et al., 2013; Froese et al., 2020), and regulation of inflammatory balance and microglial clearance of protein debris (Heneka et al., 2010; Feinstein et al., 2016; Ardestani et al., 2017; Evans et al., 2020). Recently, there has been increasing interest in the potential for restoring lost adrenergic tone in treatment of neurodegenerative diseases, including Parkinson’s disease (O’Callaghan et al., 2021), with pharmacoepidemiology studies also supporting the β-AR family as promising therapeutic targets in humans (Mittal et al., 2017; Gronich et al., 2018; Cepeda et al., 2019; Koren et al., 2019).

Detailed characterization of the functional expression of distinct β-AR populations in glial and non-glial cells is important to predict their suitability as potential therapeutic targets. Changes in receptor expression level or coupling efficiency between systems, and the resulting changes in agonist potency and maximal efficacy values, can confound translatability of agonist impact for drug development programs. Should novel, brain directed, β-AR agonist therapeutics be developable, translational methods will be crucial for comparing agonist profiles, understanding differential receptor expression across different tissues, and modeling the impact on brain function from preclinical data to clinical data.

One recent analytical advancement for the pharmacologist is the use of log(E_max_/EC_50_) (Kenakin, 2017), which has demonstrated utility in examining ligand bias across several receptor families (including G protein-coupled receptor (GPCR) and non-GPCRs) (Karl et al., 2020; Griffith et al., 2022; Reininghaus et al., 2022), as well as meta-analysis of GPCR: G protein coupling (Hauser et al., 2022). In these methods, agonist action is compared to that of a reference agonist, from which Δlog(E_max_/EC_50_) values are derived, allowing cell- or assay-specific features to be neutralized and permitting agonist activity translation across systems. Using cAMP concentration–response curves, we calculated isoprenaline-referenced Δlog(E_max_/EC_50_) values for established β-AR agonists for each of the three different β-AR subtypes (β_1_-AR, β_2_-AR, and β_3_-AR), expressed recombinantly. These data generated an “agonist fingerprint” for each β-AR subtype functionally expressed. We then used this fingerprint to assess functional expression in previously uncharacterized central nervous system (CNS) relevant cell lines, iPSC-derived human brain cell types and primary human glia. We also used Δlog(E_max_/EC_50_) to assess β-arrestin recruitment and desensitization, key components of the cycle of GPCR activation and inactivation (Luttrell and Lefkowitz, 2002).

Together, this work provides an in-depth characterization of signaling responses to β-AR agonists and further tests the use of Δlog(E_max_/EC_50_) analysis as a framework for characterizing GPCR expression and function. We demonstrate that β_2_-AR functional expression is most widespread among isolated cell types from the human brain, and our data suggest that established β-AR agonists are essentially unbiased in their activation of β_2_-AR for Gs versus β-arrestin and signal desensitization. This characterization provides an important reference for further *in vitro* functional studies of adrenoceptor activation in the human brain.

## 2 Materials and methods

### 2.1 Compound sources and handling

Compounds were sourced as follows: isoprenaline (TCI I0260), adrenaline (Sigma E4250), noradrenaline (Matrix Scientific 037592), dobutamine (Tocris 0515), prenalterol (Santa Cruz Bio sc-280023), formoterol (Apex Bio B1359), clenbuterol (Sigma C5423), salbutamol (Sigma S8260), tulobuterol (Alfa Aesar J61448), and mirabegron (Med Chem Express CS-0915). Upon arrival, ∼2 mg of the compound was weighed out and resuspended in DMSO to achieve a final concentration of 10 mM. Compounds were aliquoted and stored at −80°C or −20°C until use. A limit of five freeze/thaw cycles was implemented for each aliquot.

### 2.2 Cell culture

All cell sources, exogenous protein expression information, and growth media conditions are detailed in Supplementary Table S1. All cells were grown in a 37°C, 5% CO_2_ humidified incubator. Any CHO-K1 cell line recombinantly expressing a receptor transgene was grown in media supplemented with 1 mg/mL G418 (Sigma A1720) to maintain selection for transgene expression.

### 2.3 Cell engineering

CHO-K1 cell transgene lines engineered at CuraSen were all prepared from human codon-optimized, gene-synthesized constructs (GenScript) corresponding to sequences with protein accession numbers detailed in Supplementary Table S1. Genes were NotI/XbaI restriction digested into the pCMV6KN expression vector (OriGene) and transfected into CHO-K1 cells using Lipofectamine 3000, following the manufacturer’s instructions. Up to 1 mg/mL G418 (Sigma A1720) was supplemented to media 48 h post transfection to positively select for and maintain transfected cells.

Monoclonal cell lines were generated by dilution cloning. In brief, single cells were grown in 96-well plates, allowed to proliferate into stable cultures, and assessed for receptor expression by again assessing the responses to established receptor agonists.

To create THP-1 cells expressing only β_1_-AR or β_2_-AR, the β_2_-AR or β_1_-AR coding gene (ADRB1 or ADRB2, respectively) was knocked out using CRISPR/Cas9 (Synthego). THP-1 cells were nucleofected with optimized sgRNA/SpCas9 ribonucleoprotein complexes, with the ADRB1 or ADRB2 gRNA target sequences: GCG​GCC​CCA​CAC​CAC​GAU​GG and CGU​CUG​CAG​ACG​CUC​GAA​CU, respectively. The indel percentage in the cell population after knockout was 65% and 67%, from which monoclonal cell populations were grown in-house as previously described to identify and isolate knockout clones.

To create C6 rat glioma cells expressing only β_1_-AR, the β_2_-AR coding gene (*Adrb2*) was knocked out using CRISPR/Cas9 (Synthego). C6 cells were nucleofected with optimized sgRNA/SpCas9 ribonucleoprotein complexes with the *Adrb1* or *Adrb2* gRNA target sequence: GCU​GCU​GCC​UCC​AGC​CAG​CG and CCU​GGC​GCU​CGG​CUU​CCA​UU. The indel percentage in the cell population after knockout was 65% and 85%, from which monoclonal cell populations were grown in-house as previously described to identify and isolate knockout clones.

ReNcell VM cells expressing β_2_-AR were generated by infecting ReNcell VM cells with a lentivirus containing the ADRB2 gene (OriGene RC204499L3V) at an MOI of 12.5, and 48 h post-infection, cells were grown in media containing 0.25 μg/mL puromycin.

To passage adherent cells, cells were released either using EDTA solution (Versene, Lonza 17-711E) after washing in 1x PBS (Caisson PBL05) or using Accutase (Mediatech 25-058-CI) after washing in 1x PBS.

### 2.4 cAMP HTRF assay

cAMP accumulation was measured using the cAMP Gs Dynamic HTRF kit (Cisbio/Perkin Elmer 62AM4PEC), broadly following the manufacturer’s instructions. Compounds were prepared at a 2x final concentration to accommodate the subsequent addition of cells. To prepare compound dose–response curves, compounds were diluted to the necessary highest concentration (default 10 μM, but dependent on compound potency) in 1x stimulation buffer containing 500 μM 3-isobutyl-1-methylxanthine (IBMX) and dispensed into a 96-well U-bottom polypropylene assay plate (Corning 3365). Vehicle (DMSO), a maximally active isoprenaline dose (0.1 μM for β_1_-AR and β_2_-AR and 1 μM for β_3_-AR), and a full isoprenaline dose–response curve were added as controls on all assay plates. β-AR agonists were serially diluted across the plate by nine 5-fold dilutions to generate a 10-point dose–-response curve.

Five microliters from 96-well compound source plates were stamped into every well of a 384-well white-bottom plate (Corning 3825) using a VIAFLO 384 equipped with a 0.5–12.5 μL pipetting head (Integra), thus creating four technical replicates for each treatment condition. Plates (384-well) containing compounds were covered and stored at room temperature until addition of resuspended cells.

D2-labeled cAMP (acceptor) and europium cryptate-labeled cAMP antibody (donor) (Cisbio/Perkin Elmer 62AM4PEC) were dissolved in water following the manufacturer’s instructions, aliquoted, and stored at −80 °C. For detection of cAMP, on the day of the assay prior to addition of cells to compounds, both reagents were diluted 21-fold in the provided lysis buffer (Cisbio/Perkin Elmer), combined, and stored at room temperature prior to addition to assay plates.

Resuspended cells were resuspended in a minimal volume of 1x stimulation buffer containing 500 μM IBMX at room temperature. All cell suspensions were further diluted in 1x stimulation buffer containing 500 μM IBMX to a density corresponding to the lower values of the dynamic range of the cAMP HTRF assay, with each cell system optimized individually. Five microliters of cells of correct density were added to the compound in assay plates using a Multidrop Combi (Thermo Fisher). Cells and compounds were covered with a clear plate seal (Axygen PCR-SP) and incubated at 37°C, 5% CO_2_ for 30 min. cAMP accumulation was then stopped by adding 10 μL HTRF detection reagents in lysis buffer using the Multidrop Combi and plates were covered with an aluminum plate seal (Axygen PCR-AS-600) and agitated (Heidolph Titramax 1000, setting 600) for at least 2 h, to allow competition between D2-cAMP and stimulated cAMP for the donor-labeled cAMP antibody to reach equilibrium.

HTRF was detected using a Tecan Spark plate reader, operated using SparkControl™ software, using the TR fluorescence intensity setting. Both the donor and acceptor were excited at 320 nm, detected after 100-μs delay, with 400-μs integration time, and 50 flashes per read. Donor fluorescence was detected at 620 nm, and acceptor fluorescence was detected at 665 nm. To standardize reads across experiments, camera gain was manually set to 120 for donor detection and 140 for acceptor detection.

### 2.5 cADDis assay

cADDis fluorescence assays were performed broadly as described by the manufacturer (Montana Molecular D0200G). A single cADDis infection reaction comprised 25 μL cADDis mix (3.75 μL cADDis sensor BacMam, 0.3 μL 500 mM sodium butyrate, and 20.95 μL ‘cADDis media’ (FluoroBrite media + GlutaMAX (Gibco) + 10% FBS, 1% P/S)), combined with 25 μL cADDis media containing 1321N1 cells at a density of 6 × 10^4 cells/mL. A mixture of cADDis reaction and 1321N1 cells was prepared to provide enough volume to seed the middle 240 wells of a 384-well plate (Corning 3764). Using a VIAFILL (Integra) equipped with a 16-channel head, 50 μL of cADDis: 1321N1 cells was added to each well (1,500 cells per well), and the plate was incubated at room temperature for 15 min before overnight incubation in a 37°C, 5% CO_2_ humidified incubator.

After 24 h of incubation, cells were imaged using an Incucyte S3 (green and phase channel) to measure cADDis fluorescence intensity at baseline (unstimulated). Compounds were diluted to the 5x final concentration in cADDis media, serially diluted while maintaining vehicle (DMSO) concentration, and 12.5 uL of 5x compound was seeded into the cADDis 1321N1 cell plate using a VIAFLO 384 (Integra). The 1321N1 cells were immediately imaged again on the Incucyte (the final image time point was 10 min post drug addition).

For each well, the cADDis intensity after compound addition was normalized to the mean of the vehicle control wells, and this intensity was corrected for the cADDis intensity of that well at baseline (pre-compound addition, Time 0) as it related to the mean baseline intensity of all wells across the test plate.

### 2.6 Isoprenaline Δlog(E_max_/EC_50_) analysis and radar plot generation

β-AR agonist log(E_max_/EC_50_) values were calculated for each concentration–response curve using GraphPad Prism and Microsoft Excel. A reference agonist (isoprenaline) dose–response curve was generated in the same plate as each test agonist, on the same experimental day with identical cell and assay reagents. Therefore, β-AR agonist Δlog(E_max_/EC_50_) values, relative to isoprenaline, were also calculated on a plate-matched basis. Average isoprenaline Δlog(E_max_/EC_50_) values for each β-AR agonist were used to generate β-AR fingerprint radar plots of system responses to β-AR agonists, again using Microsoft Excel. Therefore, radar plots reflect Δlog(E_max_/EC_50_) mean values calculated from the entire dataset presented in Supplementary Table S2, which contains both tandem- (different test agonists evaluated on different experimental days) and parallel-design (panel of agonists assayed on the same experimental day) experiments, but where each Δlog(E_max_/EC_50_) value is calculated on a plate-matched basis.

A comparison between Δlog(E_max_/EC_50_) error calculations using tandem versus parallel experimental design is shown in Supplementary Table S3. The first method is identical to that shown for the full dataset in Supplementary Table S2, reflecting the nature of plate-matched Δlog(E_max_/EC_50_) values, and the second method follows that described in Kenakin et al. (2012). The latter method is included to inform any uses of this reference dataset in an experimental design, which would not allow technical matching of test and reference agonists.

### 2.7 DiscoverX PathHunter^®^ arrestin recruitment assay

DiscoverX PathHunter^®^ cells expressing β_2_-AR (93–0182C2) with a receptor ProLink™ tag and arrestin enzyme acceptor tag were cultured in T75 flasks at 37°C, 5% CO_2_ in a growth medium containing DMEM/F12 (Caisson Labs DFL13), 10% FBS (Corning 35-010-CV), 1x penicillin/streptomycin (Caisson Labs PSL01), 1 mg/mL G-418 (Caisson Labs G030), and 300 μg/mL hygromycin (Caisson Labs H010) and were passaged before full confluence. Cells were seeded in 384-well plates (Corning 3570) at a density of 5,000 cells per well in 20 μL growth medium. The next day, solutions of β-AR agonist were prepared as 5-fold serial dilutions in a 96-well plate (Corning 3363) at the desired final concentration of 5x. About 5 μL of the drug solution was transferred from this 96-well source plate to the 384-well cell plate using a VIAFLO 384 equipped with a 0.5–12.5 μL pipetting head (Integra), generating four technical replicates for each drug dose. Plates were sealed with clear plastic (Corning AXYPRSP) and incubated for 90 min in a 37°C, 5% CO_2_ incubator. Meanwhile, the working detection solution (WDS) was freshly prepared at room temperature from kit reagents (93-0001L) as per the manufacturer’s instructions in a 19:5:1 ratio (buffer: component 1: component 2). After drug incubation was complete, 12.5 μL WDS was added per well using a Multidrop Combi. Plates were covered with an aluminum plate seal (Axygen PCR-AS-600) and agitated (Heidolph Titramax 1000, setting 600) for 90 min. Luminescence was detected (360–700 nm) with a Tecan Spark plate reader, operated using SparkControlTM software, using 500 ms integration time.

### 2.8 Functional desensitization assay

1321N1 cells were seeded in 384-well plates (Corning 3570 or 3764) at a density of 5,000 cells per well in 40 μL growth medium. The next day, solutions of β-AR agonists were prepared as 5-fold serial dilutions in a 96-well plate to the desired final concentration of 5x. About 10 μL of the drug solution was transferred from the 96-well source plate to the 384-well cell plate using a VIAFLO 384 equipped with a 0.5–12.5 μL pipetting head (Integra), generating four technical replicates for each drug dose. A full concentration–response curve of isoprenaline was prepared for each cell plate to allow in-plate determination of Δlog(E_max_/EC_50_). Cells were incubated with 1x drug solution for 24 h. After 24 h of incubation, cells were washed three times by inverting and gently tapping plates onto paper towels, followed by centrifugation of the inverted plate at 30 x g, before adding 40 μL of wash solution containing DPBS, + 0.1% bovine serum albumin (VWR 97061-416) using a VIAFILL dispenser (Integra). After the third wash solution removal, 10 μL of a solution of HTRF Stimulation Buffer 1 (Cisbio/Perkin Elmer 64SB1FDC) containing 500 μM IBMX (Cayman 13347) and 5 μM tulobuterol was added to each well with a VIAFLO pipette. The cell plate was sealed with clear plastic and incubated at 37°C for 30 min. D2-labeled cAMP (acceptor) and europium cryptate-labeled cAMP antibody (donor) (Cisbio/Perkin Elmer 62AM4PEC) were dissolved in water and added to cell plates as previously described for the cAMP HTRF assay, followed by similar signal detection using a Tecan Spark plate reader.

### 2.9 Data and statistical analysis

Concentration–response curves shown in figures display mean ± SEM across n = 4 technical replicates from a single representative dose–response curve. For dose–response curves generated from cAMP HTRF, raw ratiometric HTRF signals, relative to the maximum effect of isoprenaline, were plotted, unless stated. Each compound was assayed a minimum of three times in independent experiments. The exact experimental replicate number is shown in Supplementary Table S2. Correlation plots (Figures 6 and 7) show mean Δlog(E_max_/EC_50_) values ± 95% CI across n = 3 independent experiments.

cAMP HTRF ratios were obtained by applying the formula

$$\left(Abs\left(665\;nm\right)/Abs\left(620\;nm\right)\right)\;x\;10,000,$$

where Abs (665 nm) and Abs (620 nm) are the absolute fluorescence units detected at 665 nm (HTRF acceptor) and 620 nm (HTRF donor), respectively.

For all dose–response curves generated across all assay readouts (cAMP HTRF and cADDis), the potencies of test and control compounds were determined by non-linear regression using GraphPad Prism. HTRF ratios, from quadruplicate measures at each concentration, were plotted versus the log concentration of β-AR agonist and analyzed using the following 4-parameter logistic equation:

$$y=Bottom+\frac{x^{Hill\;slope}\cdot \left(Top-Bottom\right)}{x^{Hill\;slope}+{{EC}_{50}}^{Hill\;slope}}.$$ (1)

All dose–response curves on a given plate were simultaneously analyzed to define a single, shared baseline value, and the Hill slope was constrained to be >0, to avoid false curve fits from inactive compounds. The average (mean) β-AR agonist potency (pEC_50_) and maximum effect (E_max_) relative to the within-plate isoprenaline dose response were reported.

For normalizing cAMP HTRF ratiometric data to a cAMP standard curve, cAMP (Sigma A6885) was dissolved, and a concentration range was prepared by serial dilution in 1x stimulation buffer. The cAMP standard curve was detected by cAMP HTRF contemporaneously with β-AR agonist-stimulated 1321N1 cells, as described previously. The cAMP standard curve was fitted to a non-linear regression 4-parameter logistic equation as described previously, with a separate baseline value from β-AR agonist dose responses in cells. The standard curve was used to interpolate per-well cAMP quantities from raw ratiometric cAMP HTRF values for each dose of each β-AR agonist. β-AR agonist cAMP-normalized concentration dose–response curves were then analyzed using 4-parameter logistic regression, as described previously, to assess the potency and efficacy of compounds.

Representative dose–response curves shown were compiled from raw cAMP HTRF ratiometric values, first by selecting individual agonist dose responses that closely resembled average potency and maximal efficacy values. Those selected datasets were normalized to the plate-matched isoprenaline dose–response curve, first by calculating the individual cAMP HTRF ratio value relative to the average of the isoprenaline baseline (lowest concentration isoprenaline value) and then by dividing that value by the average maximal (highest concentration isoprenaline value) isoprenaline response to make the isoprenaline dose–response curve 100%.

To assess β-AR agonist selectivity at β_1_-AR versus β_2_-AR, average β-AR agonist Δlog(E_max_/EC_50_) values, relative to isoprenaline, for β_2_-AR (in CHO-K1 cells) were subtracted from the average β-AR agonist Δlog(E_max_/EC_50_) values, relative to isoprenaline, for β_1_-AR (again in CHO-K1 cells) to generate ΔΔlog(E_max_/EC_50_) values (Supplementary Table S4). This analysis was performed on a subset of six experiments run in parallel, where all agonists and cell lines displayed were tested on the same experimental day. ΔΔlog(E_max_/EC_50_) values were also calculated for cAMP, arrestin recruitment, and functional desensitization assays (Supplementary Table S4). 95% confidence intervals were calculated according to the method described in Kenakin et al. (2012) and Servant et al. (2022).

## 3 Results

### 3.1 Recombinant cell systems define agonist fingerprints for β-AR subtype functional expression

We first established a reference agonist activity dataset in recombinant β-AR expression systems. The activity of β-AR agonists was measured by detecting cAMP increases in CHO-K1 cells expressing either β_1_-, β_2_-, or β_3_-AR human homologs. We characterized the endogenous β-AR agonists adrenaline and noradrenaline, the potent and non-selective β_1_/β_2_-AR agonist isoprenaline, β_1_-AR selective agonists dobutamine and prenalterol, β_2_-AR selective agonists salbutamol, clenbuterol, tulobuterol, and formoterol, and the β_3_-AR selective agonist mirabegron. As a negative control, CHO-K1 cells lacking recombinant expression showed no cAMP response to any agonist tested (Supplementary Figure S1).

**FIGURE 1:**
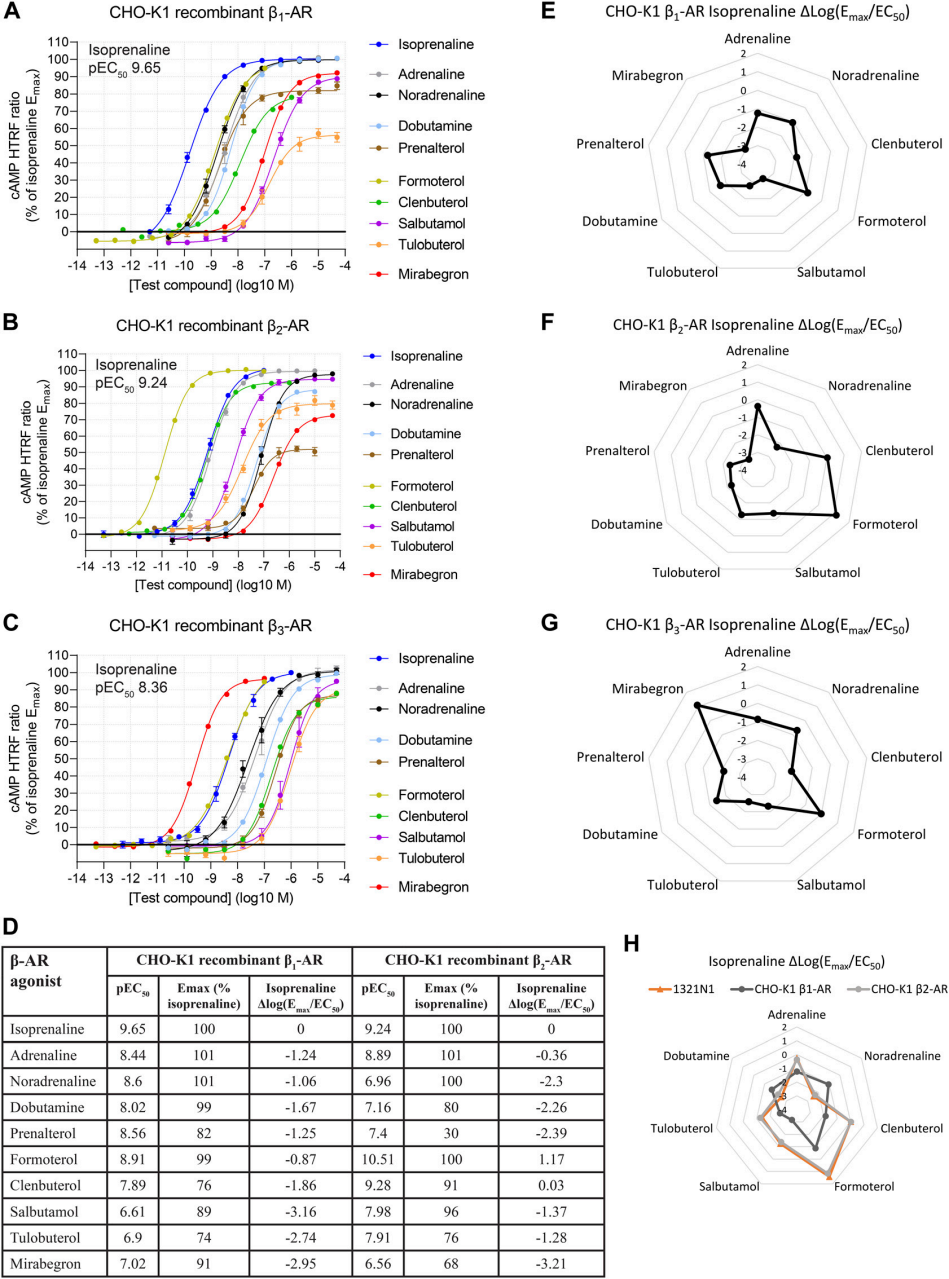
Functional cAMP responses to a panel of β-AR agonists in CHO-K1 cell lines recombinantly expressing **(A)** β_1_-AR, **(B)** β_2_-AR, or **(C)** β_3_-AR human receptors. Representative curves are displayed, each with four technical replicates, showing mean ± SEM cAMP HTRF responses normalized to the full agonist isoprenaline. Each agonist curve was converted to a Δlog(E_max_/EC_50_) agonist fingerprint using isoprenaline as the reference molecule **(D–G)**. This allows a system-independent visualization of agonist similarity to isoprenaline (nodes) and receptor expression (edges) across a range of isoprenaline potencies. A similar analysis in the endogenous expression system 1321N1 astrocytoma cell line reveals an agonist fingerprint, consistent with β_2_-AR and not β_1_-AR expression **(H)**. Average concentration–response curves for β_1_-AR and β_2_-AR across a subset of six experiments with agonists tested in parallel are shown in Supplementary Figure S2.

For each concentration–response curve, ratiometric cAMP HTRF values were normalized to the maximal response of isoprenaline, which was selected as the reference full agonist throughout our study. Representative dose–response curves of β-AR activation in CHO-K1 cells and summary data are shown in Figures 1A–C, and summary data are shown in Figure 1D. β-AR activation was observed at all three β-AR subtypes in response to all β-AR agonists tested.

For each agonist in each replicate experiment, we calculated the log_10_-transformed E_max_/EC_50_ ratio (Equation 2: Emax as a fraction of the reference agonist; EC_50_ expressed in M units). We then subtracted the same metric of the plate-matched reference agonist, isoprenaline, for which E_max_ is defined as 1.

$$∆\log\left(\frac{E_{\max}}{{EC}_{50}}\right)={\log\left(\frac{E_{\max}}{{EC}_{50}}\right)}_{test\;agonist}-{\log\left(\frac{E_{\max}}{{EC}_{50}}\right)}_{isoprenaline}$$ (2)

The resulting value, hereby referred to as Δlog(E_max_/EC_50_), provides a system-independent metric for agonist activity relative to isoprenaline (Kenakin, 2017), provided Hill slopes are near unity (Winpenny et al., 2016).

Δlog(E_max_/EC_50_) is, in theory, independent of tissue, assay, and receptor coupling, as variations in receptor expression, amplification, or other variables are canceled by the comparison to a reference agonist (Kenakin, 2017). Accordingly, Δlog(E_max_/EC_50_) values across a panel of agonists provide a composite readout of receptor activation by those agonists as a profile. Δlog(E_max_/EC_50_) values were arranged in a radar plot to give each β-AR population a distinct visual agonist fingerprint (Figures 1E–G for the three recombinant human β-ARs), which could then be compared across other cell system assays. In this agonist fingerprint, values further from the center represent greater activity, while values closer to the center represent lower activity, all relative to isoprenaline, for which the Δlog(E_max_/EC_50_) is 0. The exact shape of the agonist fingerprint is arbitrary, as it depends on the agonist arrangement on the radar axes. Imposing a fixed orientation of agonists on the plot, the shape of the fingerprint is unique for each receptor subtype. The resulting Δlog(E_max_/EC_50_) β-AR agonist fingerprints allow functional expression of the three β-ARs to be easily distinguished from one another.

Δlog(E_max_/EC_50_) analysis may also be used to analyze selectivity for any of the test agonists for one receptor subtype over another, by comparing Δlog(E_max_/EC_50_) values across two assay systems (ΔΔlog(E_max_/EC_50_)) (Kenakin, 2017). Interpretation of selectivity using ΔΔlog(E_max_/EC_50_) is relative to the reference agonist, so it is optimal when that reference agonist is thought to be non-selective between receptors being examined. As isoprenaline is thought to be non-selective at β_1_-AR vs. β_2_-AR (Baker, 2010), we examined β_1_-AR vs. β_2_-AR selectivity using ΔΔlog(E_max_/EC_50_) (Supplementary Figure S2) and found selectivity consistent with that of previous reports of Baker (2010). We did not assess selectivity involving β_3_-AR, for which isoprenaline has lower potency (Baker, 2010). For this reason, and because we did not observe a β_3_-AR fingerprint in all other cell lines described as follows, β_3_-AR (and the agonist mirabegron) are not further discussed here.

### 3.2 Using agonist fingerprinting to define β-AR expression in native cell systems expressing single or multiple β-AR populations

The utility of Δlog(E_max_/EC_50_) agonist fingerprinting was assessed in a native expression system, 1321N1 astrocytoma cells: a human cell line reported to express β_2_-AR (Su et al., 1979; Doss et al., 1981). β-AR agonist-driven cAMP responses were readily detectable in 1321N1 cells (Supplementary Figure S4D). β-AR agonist potency and E_max_ values in 1321N1 cells were different from the potency and E_max_ values measured in all CHO-K1 recombinant systems (Supplementary Table S2), consistent with decreased receptor reserve for the native cell-line versus recombinant cells.

We represented β-AR agonist activity in 1321N1 cells as a Δlog(E_max_/EC_50_) β-AR agonist fingerprint (Figure 1H). Agonists showing no response or incomplete curves were removed as axes in the radar plot. The 1321N1 β-AR agonist fingerprint matched that observed in the CHO-K1 recombinant system expressing β_2_-AR, suggesting that 1321N1 cells indeed naturally express β_2_-AR. We confirmed this result using selective β-AR antagonists, observing that the β_2_-AR selective antagonist ICI-118,551 displayed a 10^5^-fold lower inhibition constant (IC_50_) versus the β_1_-AR selective antagonist CGP-20712A (Supplementary Figure S3). These data confirm that 1321N1 cells natively express β_2_-AR and validate, with real-world data, the use of Δlog(E_max_/EC_50_) as a method of assessing β-AR subtype functional expression independent of receptor expression density or coupling efficiency.

To test the performance of Δlog(E_max_/EC_50_) agonist fingerprinting using alternative cAMP detection assays, we employed cADDis (Montana Molecular), a live-cell fluorescent biosensor that decreases in fluorescence intensity when bound to cAMP (Tewson et al., 2016). We measured β-AR agonist activity in 1321N1 cells expressing cADDis and observed an agonist-mediated, dose-dependent decrease in cADDis fluorescence (Supplementary Figures S4A, B). The cADDis assay was less sensitive than cAMP HTRF detection methods, which limited the panel of β-AR agonists for which we could generate full dose–response curves. Nevertheless, measurable Δlog(E_max_/EC_50_) β-AR agonist values from cADDis matched Δlog(E_max_/EC_50_) values derived from cAMP HTRF, and therefore generated an identical β-AR agonist fingerprint to that derived from cAMP HTRF (Supplementary Figure S4C). These data confirm that Δlog(E_max_/EC_50_) activity determination is agnostic of the reagent used to measure agonist activation of cyclic AMP production.

Lastly, to ensure that normalization methods (Burford et al., 2017) do not affect the interpretation of Δlog(E_max_/EC_50_), we confirmed that dose–response curves derived from HTRF values pre- and post-normalization by a cAMP standard curve gave identical Δlog(E_max_/EC_50_) values (Supplementary Figures S4D–F). This finding supports the derivation of Δlog(E_max_/EC_50_) from ratiometric HTRF data, minimizing data transformation steps.

**FIGURE 2:**
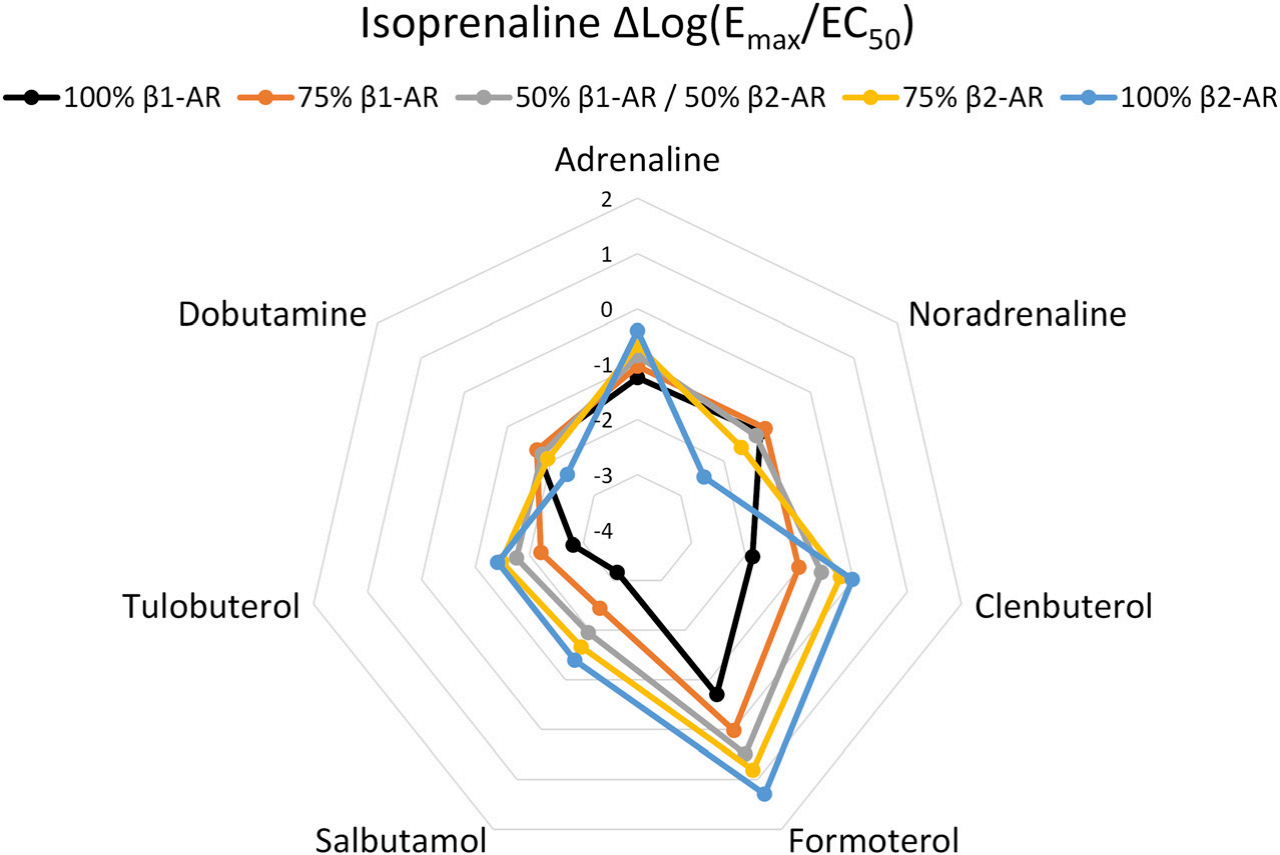
Agonist fingerprint derived from functional cAMP curves following admixture of CHO-K1 cells recombinantly expressing either β_1_-AR or β_2_-AR human receptors. Mixed-expression systems display fingerprints with hybrid features relative to either of the pure expression system.

To determine whether agonist fingerprinting could be useful in systems with expression of multiple receptors, we examined how Δlog(E_max_/EC_50_) values are affected when more than one β-AR is present. First, we mixed CHO-K1 cells recombinantly expressing either β_1_-AR or β_2_-AR, at defined ratios of seeding density, and assessed the combined cAMP response. Mixed CHO-K1 β-AR populations produced intermediate agonist fingerprints versus pure expression populations (Figure 2; Supplementary Figure S5; Supplementary Table S2). These data highlight that the Δlog(E_max_/EC_50_) method of analysis can detect mixed-receptor expression among cell populations.

**FIGURE 3:**
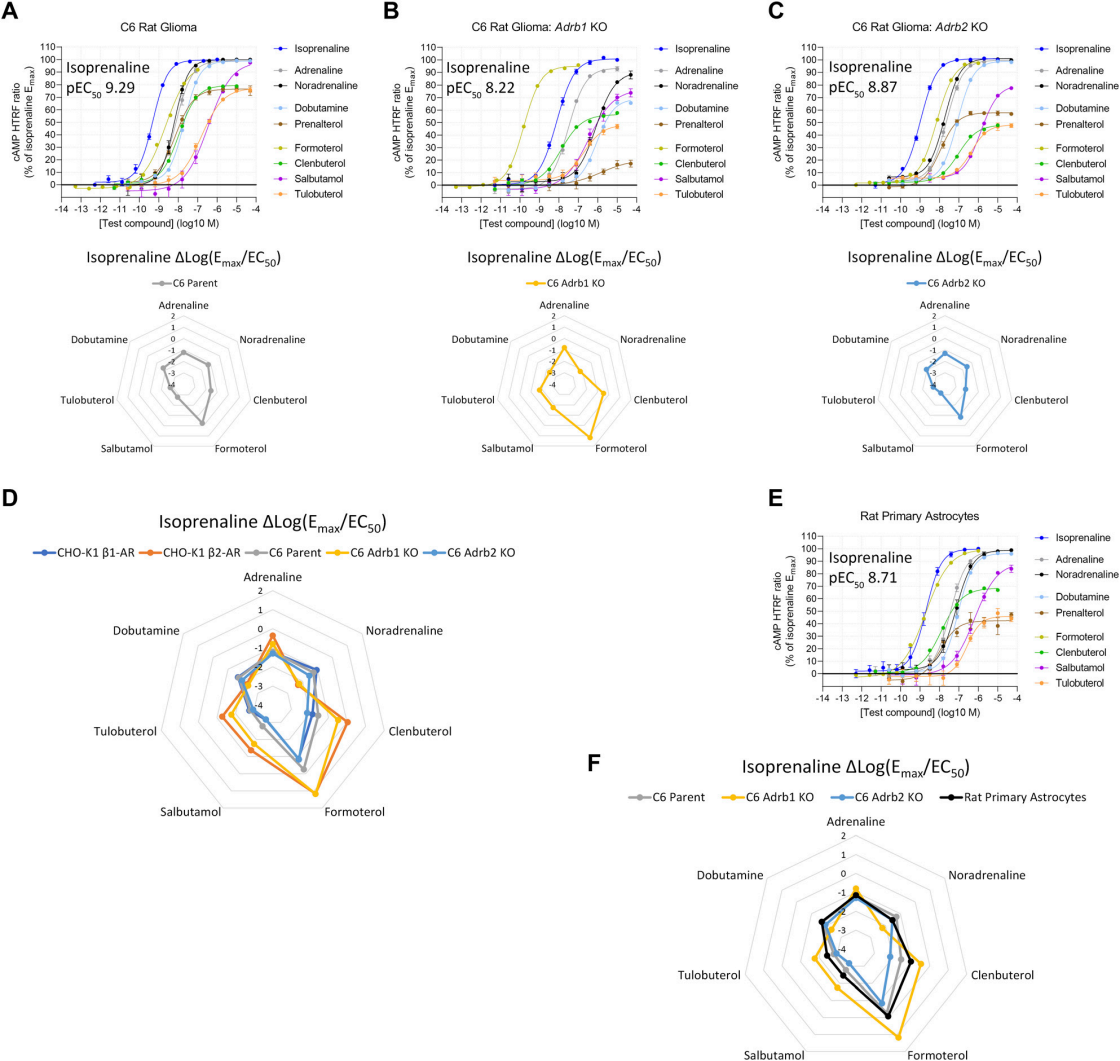
Functional cAMP responses to a panel of β-AR agonists in the C6 rat glioma cell line with **(A)** native expression, **(B)** *Adrb1* (β_1_-AR) knockout, or **(C)** *Adrb2* (β_2_-AR) knockout. Representative curves are displayed, each with four technical replicates, showing mean ± SEM cAMP HTRF responses normalized to the full agonist isoprenaline. Each agonist curve was converted to a Δlog(E_max_/EC_50_) agonist fingerprint using isoprenaline as the reference molecule. Radar plots below the concentration–response curves demonstrate an agonist fingerprint of the C6 cell line non-overlapping with human β_1_-AR or β_2_-AR receptors, suggesting possible species differences and/or dual-receptor expression. **(D)** Overlay of agonist fingerprints across species for single-receptor systems from either rat (C6 knockout) or human (CHO-K1 recombinant), with an intermediate profile from the C6 co-expressing cell line. **(E)** Rat primary astrocytes display agonist responses and an agonist fingerprint with hybrid features **(F)**, indicating endogenous co-expression of β_1_-AR and β_2_-AR.

We then applied this finding to two rat brain cell types which responded to β-AR agonists, but which showed distinctly different agonist fingerprints. First, C6 cells, a rat glioma cell line (Benda et al., 1968), produced a Δlog(E_max_/EC_50_) β-AR agonist fingerprint that did not correspond to either exclusively human β_1_-AR or human β_2_-AR expression profiles (Figure 3A). To test for expression of multiple β-AR subtypes, we used CRISPR/Cas9 to knockout (KO) either *Adrb1* or *Adrb2* (the rat β_1_-AR or β_2_-AR genes, respectively) and reassessed functional β-AR expression. C6 *Adrb1* KO cells (Figure 3B) showed a β_2_-AR fingerprint, and C6 *Adrb2* KO cells (Figure 3C) showed a β_1_-AR-like fingerprint (Figure 3D). Agonist activity differences were detected between rat and human ARs, potentially illustrating the species-dependence of the Δlog(E_max_/EC_50_) metric, as expected for AR homologs of different primary sequences and agonist pharmacology (Strasser et al., 2013; Supplementary Table S2). Mixed β_1_-AR and β_2_-AR expression was also identified in rat primary cortical astrocytes (Figures 3E, F). These examples demonstrate that endogenous mixed-receptor expression within a single cell type can be identified using an agonist fingerprinting method and supports previous *in vivo* findings suggesting dual expression of rat brain β_1_-AR and β_2_-AR (Rainbow et al., 1984). Notably, increased β_1_-AR subtype expression vs. β_2_-AR in rat brain compared to the human brain has been reported (Reznikoff et al., 1986; Joyce et al., 1992).

**FIGURE 4:**
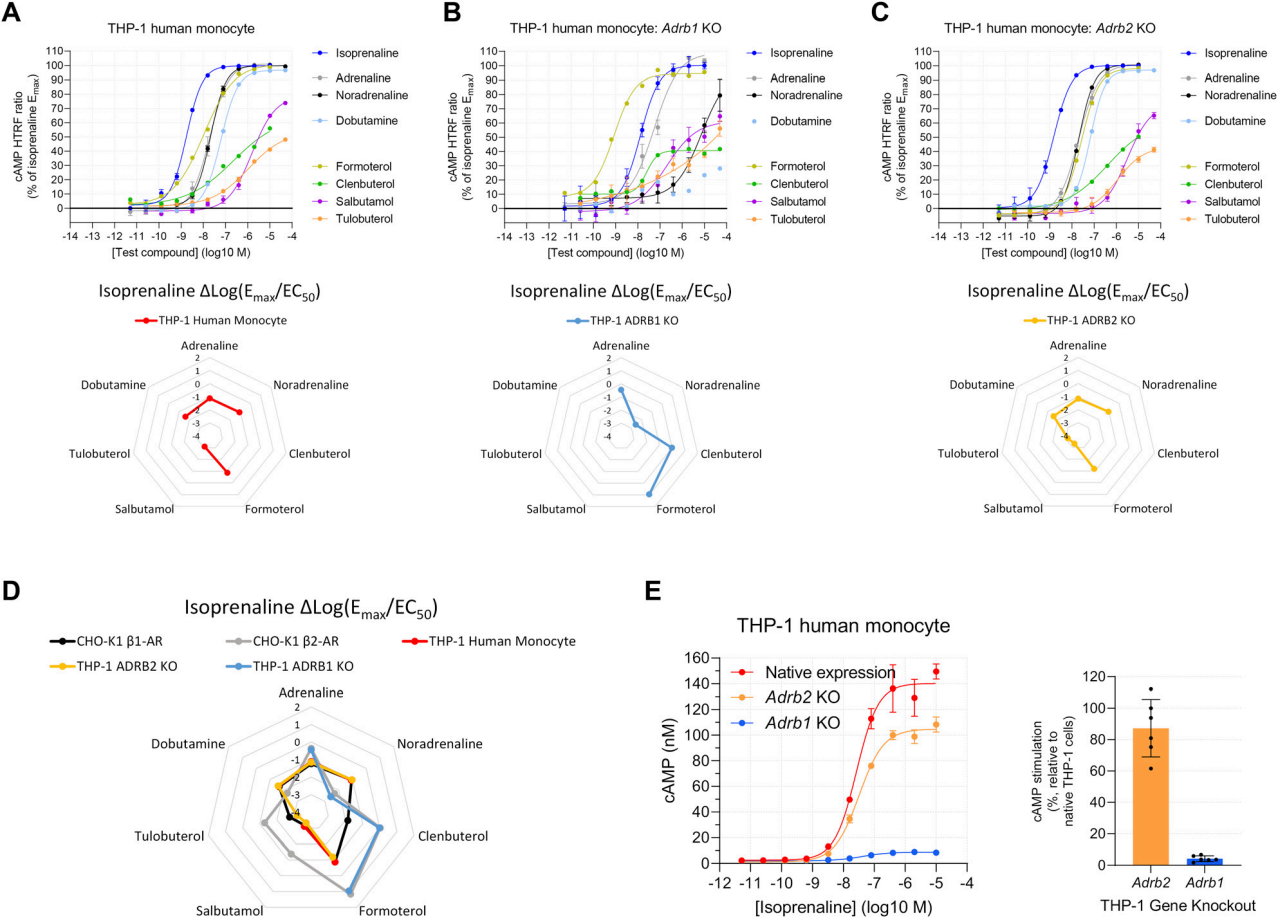
Functional cAMP responses to a panel of β-AR agonists in the human THP-1 cell line with **(A)** native expression, **(B)** *Adrb1* (β_1_-AR) knockout, or **(C)** *Adrb2* (β_2_-AR) knockout. Representative curves are displayed, each with four technical replicates, showing mean ± SEM cAMP HTRF responses normalized to the full agonist isoprenaline. Each agonist curve was converted to a Δlog(E_max_/EC_50_) agonist fingerprint using isoprenaline as the reference molecule. Radar plots below the concentration–response curves reveal an agonist fingerprint of the THP-1 cell line, suggestive of predominantly β_1_-AR function. **(D)** Overlay of agonist fingerprints across human cell lines with either endogenous or exogenous receptor expression shows good agreement for single-receptor systems. **(E)** Gene knockout of individual receptors in the THP-1 cell line confirms that the functional cAMP response in parental THP-1 cells is driven predominantly by β_1_-AR receptors; the left subpanel shows cAMP concentration after normalization of the HTRF response.

THP-1 human monocytes are reported to express either β_1_-AR (Talmadge et al., 1993) or β_2_-AR (Wang et al., 2015; Grisanti et al., 2016; Noh et al., 2017). In contrast to 1321N1 cells, we observed a β-AR agonist response in THP-1 human monocytes, suggestive of β_1_-AR expression (Figure 4A). However, a reproducibly shallow Hill slope of formoterol (mean of 0.73, Supplementary Table S2), clenbuterol (0.51), and tulobuterol (0.59), β_2_-AR selective agonists, prevented complete agonist fingerprinting (Figure 4A, lower panel) and increased the possibility of multiple β-AR subtypes. To test this possibility, we knocked out expression of either the ADRB1 or ADRB2 genes. In ADRB1 KO THP-1 cells, β-AR agonist activity consistent with β_2_-AR expression was detected (Figure 4B). ADRB2 KO THP-1 cells displayed activity consistent with β_1_-AR expression, and the average Hill slope of formoterol increased to near unity (1.04) (Figure 4C). Superimposing THP-1 and CHO-K1 Δlog(E_max_/EC_50_) β-AR agonist fingerprints confirmed functional expression of the respective receptors in KO lines (Figure 4D). We noted that, unlike formoterol, the average Hill slopes for clenbuterol (0.53) and tulobuterol (0.78) remained shallow in ADRB2 KO THP-1 cells, perhaps indicating detection of efficacy at two purportedly distinct sites of β_1_-AR (Baker, 2005; Baker et al., 2014).

The close alignment between β-AR agonist fingerprints from THP-1 and ADRB2 KO THP-1 cells suggested that β_1_-AR contributes the majority of overall cAMP production. Indeed, comparison of the cAMP response to isoprenaline between native (unmodified), ADRB1 KO, and ADRB2 KO THP-1 cells revealed THP-1 ADRB1 KO cells retain only 5% ± 2% of the cAMP response observed in native THP-1 cells (Figure 4E). In contrast, THP-1 ADRB2 KO cells retain most of their cAMP response (87% ± 18%) (Figure 4E). These data highlight the predominant expression of β_1_-AR over β_2_-AR in native THP-1 cells. Although functional β_2_-AR expression is low in THP-1 cells, the ADRB1 KO THP-1 system demonstrates that, with sufficient response detected, Δlog(E_max_/EC_50_) values remain consistent in low-expression systems.

### 3.3 β_2_-AR is expressed across a wide range of human brain cell types

**FIGURE 5:**
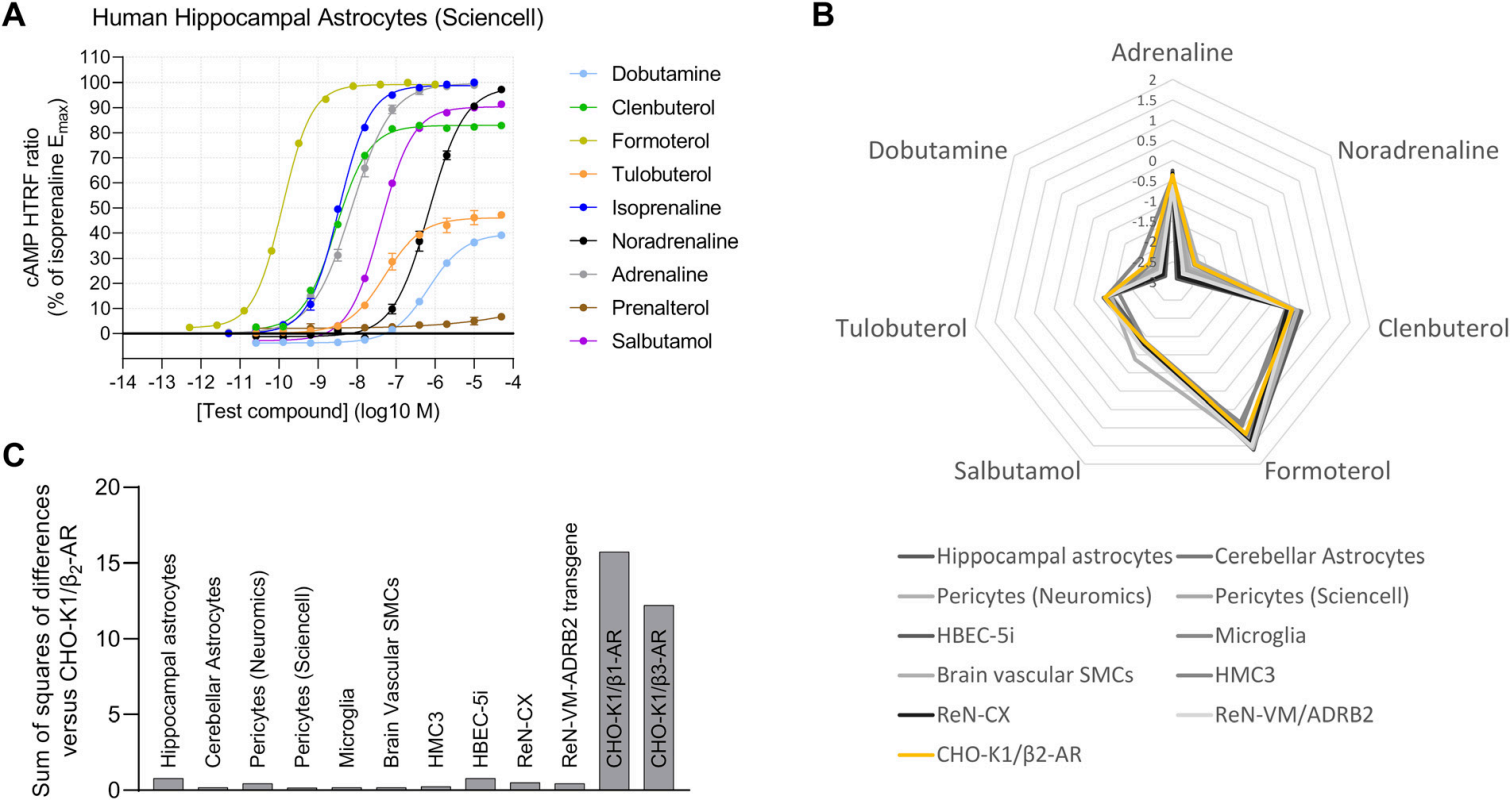
Human brain-derived cells display predominantly β_2_-AR activity. **(A)** Functional cAMP responses to a panel of β-AR agonists in primary human hippocampal astrocytes, a representative concentration–response curve from examination of several types of brain-derived human primary cells and cell lines. Representative curves are displayed, each with four technical replicates, showing mean ± SEM cAMP HTRF responses normalized to the full agonist isoprenaline. **(B)** The human brain cells tested have agonist fingerprints like those of recombinant CHO-K1/β_2_-AR cells, suggestive of predominantly β_2_-AR endogenous function. **(C)** Similarity of each agonist fingerprint to the CHO-K1/β_2_-AR system by summing the squares of differences for each agonist Δlog(E_max_/EC_50_) value.

After validating the agonist fingerprinting method across a range of expression levels and mixed-receptor systems, we used it to characterize β-AR expression across human cell systems of cerebral origin. We observed functional β_2_-AR expression in primary astrocytes isolated from either the cortex or the hippocampus (Figure 5A), primary pericytes from two commercial sources, primary brain vascular smooth muscle cells, and iPSC-derived microglia (Supplementary Figure S6A). Two human immortalized cell lines, cerebral microvascular endothelial cells (HBEC-5i) and microglia (HMC3), also expressed functional β_2_-AR (Supplementary Figure S6B), as did a neural progenitor cell line derived from the human cortex (ReNcell CX) (Supplementary Figure S6C). In contrast, neural progenitor cells derived from the ventral mesencephalon (ReNcell VM) did not naturally express high enough levels of functional β-AR to characterize the response but did show a robust β_2_-AR response when stably expressing an ADRB2 transgene (Supplementary Figure S6D), highlighting that β-AR second-messenger machinery is functional. When comparing agonist fingerprints, we observed β_2_-AR expression to be the predominant functional β-AR subtype in the range of human brain cells studied (Figures 5B, C).

### 3.4 ΔLog(E_max_/EC_50_) measurements in β-arrestin recruitment assay reveal no evidence for β_2_-AR ligand bias

**FIGURE 6:**
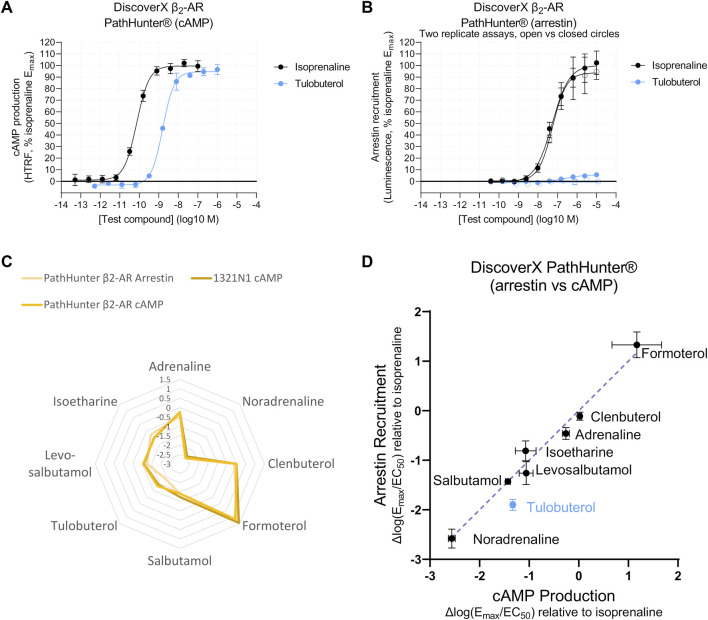
Agonist fingerprints comparing distinct signaling pathways suggest that several β_2_-AR agonists are unbiased. **(A)** Functional Gs-coupled cAMP production response to full (isoprenaline) and partial (tulobuterol) agonists in PathHunter^®^ β_2_-AR cells. Representative curves are displayed, each with four technical replicates, showing mean ± SEM cAMP HTRF responses normalized to the full agonist isoprenaline. **(B)** Arrestin recruitment in PathHunter^®^ β_2_-AR cells shows a right-shifted isoprenaline potency and decreased tulobuterol E_max_, consistent with reduced sensitivity for the arrestin-coupled readout, which leads to poor consistency in the response for weaker agonists such as tulobuterol (two example replicate curves are shown in open versus closed circles to demonstrate day-to-day variability). Summary data of additional replicates are shown in Supplementary Table S2. **(C)** Overlay of agonist fingerprints reveals similarities between arrestin recruitment and cAMP production measured in the same, or in an endogenous, expression system. **(D)** To compare just two signaling pathways, a linear correlation in Δlog(E_max_/EC_50_) values shows potential signaling bias as a deviation from the line of identity. Calculations of ΔΔlog(E_max_/EC_50_) bias factors are shown in Supplementary Table S4.

A system-independent output allows meaningful interpretation across two signaling pathways, in assays with differing sensitivity. We observed a cAMP response caused by isoprenaline and tulobuterol in CHO-K1 cells expressing modified proteins designed to measure β-arrestin recruitment (Zhao et al., 2008; DiscoverX PathHunter^®^; Bassoni et al., 2012) (Figure 6A). In the same cell line, β-arrestin recruitment for isoprenaline and tulobuterol was comparatively lessened, displaying a right-shifted isoprenaline potency (pEC_50_ = 7.3 ± 0.2), and lower E_max_ for the partial agonist tulobuterol (Figure 6B). These shifts could indicate decreased assay sensitivity for the β-arrestin readout or could reflect preferential signaling toward one pathway over another. To test this, we transformed agonist response parameters to Δlog(E_max_/EC_50_) and observed a similar agonist fingerprint with tulobuterol as an outlier (Figure 6C). As Δlog(E_max_/EC_50_) removes system-dependent effects, deviation from the line of identity in a plot of Δlog(E_max_/EC_50_)_arrestin_ vs. Δlog(E_max_/EC_50_)_cAMP_ represents ligand bias (Andresen, 2011; Rajagopal et al., 2011; Reiter et al., 2012; Ippolito and Benovic, 2021). In a panel of eight β-AR agonists, seven fell upon the line of identity (Figure 6D), which represents a bias factor (ΔΔlog(E_max_/EC_50_) (Rajagopal et al., 2011; Winpenny et al., 2016) of 0. These data suggest these β-AR agonists are unbiased at β_2_-AR. The exception was tulobuterol, which is discussed as follows.

The cAMP measurements in PathHunter^®^ cells produced concentration–response curves with Hill slopes greater than 1 (Supplementary Table S2), and, due to this, we ensured that these observations were not influenced by receptor and β-arrestin protein modifications in the PathHunter^®^ cell system. We therefore tested the correlation of Δlog(E_max_/EC_50_)_arrestin_ (PathHunter™) with Δlog(E_max_/EC_50_)_cAMP_ values obtained from 1321N1 cells, which naturally express β_2_-AR (Figure 1H; Supplementary Figures S3, S4). The same seven agonists fell on the line of identity when correlating β-arrestin response with cAMP derived from 1321N1 cells (Supplementary Figure S7B). This corroborated our finding in the PathHunter^®^ cell line, indicating that PathHunter^®^ and 1321N1 cells display similar β_2_-AR-G coupling (Supplementary Figure S7), and suggests that the β-AR agonists tested do not display evidence of ligand bias at β_2_-AR in terms of cAMP stimulation vs. β-arrestin recruitment.

As previously mentioned, the one exception in the PathHunter^®^ data was tulobuterol, which did not fall on the line of identity (Figure 6D). However, tulobuterol was the lowest maximal efficacy compound for which a β-arrestin recruitment curve could be detected, though, in some experimental replicates, a response was barely detectable (Figure 6B). This highlights a significant limitation of the low sensitivity of β-arrestin assays for detecting responses to weaker partial agonists.

### 3.5 Functional desensitization is a more sensitive β-arrestin readout applicable to endogenous systems

**FIGURE 7:**
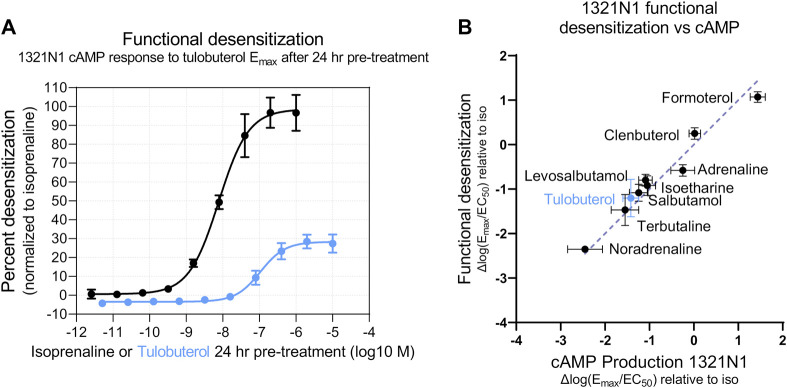
Partial agonists desensitize partially in an endogenous expression system. **(A)** Functional desensitization of cAMP production response in 1321N1 cells was calculated by measuring the cAMP produced by a single, fixed concentration of agonist after a 24-h desensitization period of incubation with test agonists across a range of concentrations. Percent desensitization of the partial agonist tulobuterol was normalized to the degree of desensitization induced by the full agonist isoprenaline. Compared with a different arrestin pathway readout in Figure 6, this functional desensitization assay shows a more potent isoprenaline response and a higher maximal tulobuterol response. **(B)** Along the line of identity in a Δlog(E_max_/EC_50_) correlation plot, tulobuterol appears as an unbiased agonist when tested in an arrestin functional assay with higher sensitivity. Calculations of ΔΔlog(E_max_/EC_50_) bias factors are shown in Supplementary Table S4.

To test whether the apparent ligand bias of tulobuterol was an artifact of low E_max_, we used an alternate method to assess presumed β-arrestin-mediated activity: functional β-AR desensitization. Functional β-AR desensitization detects a reduced cAMP response in cells pre-treated with β-AR agonists. We pre-treated 1321N1 cells in a dose-response manner with a panel of β-AR agonists (Supplementary Table S2) and, 24 h later, challenged the same cells with a single E_max_ concentration of tulobuterol. The level of cAMP produced by an E_max_ dose is suggested to be proportional to the number of active receptors (Su et al., 1979; Doss et al., 1981); thus, the second agonist treatment ostensibly allows a readout of the remaining active receptors. In this assay, pre-incubated ligands may lead to a desensitized system (most likely via β-arrestin recruitment) that is then less responsive to further agonist stimulation. Accordingly, we observed that β-AR agonist pre-treatment reduced cAMP production upon second agonist stimulation, in a dose-dependent manner. We can define functional desensitization Δlog(E_max_/EC_50_) values using test β-AR agonist desensitization curves expressed as a percentage of isoprenaline-induced desensitization (Figure 7A). Thus, Δlog(E_max_/EC_50_) desensitization values were plotted against Δlog(E_max_/EC_50_)_cAMP_ values, all measured in 1321N1 cells. In agreement with β-arrestin recruitment (PathHunter^®^) results, we observed that most tested β-AR agonists fell on the line of identity, suggesting these β-AR agonists are unbiased (Figure 7B). Because the functional desensitization assay is more sensitive (compare tulobuterol response in Figure 7A; Figure 6B), tulobuterol gives improved detection of maximal response, falling on the line of identity (Figure 7B), and thus also appears unbiased.

Taken together, our system-independent analysis demonstrates that partial β-AR agonists for cAMP production also show partial agonism for β-arrestin recruitment. Partial β-AR agonists also cause a partial degree of functional desensitization. The panel of agonists presented here display varying strengths in potency and intrinsic activity in both assays, and the use of Δlog(E_max_/EC_50_) transformation indicates a lack of ligand bias.

## 4 Discussion

The ability to characterize GPCR expression in systems with native levels of expression, transducer coupling, and effector signaling enables robust lead optimization for GPCR drug discovery programs. Here, we present a radar plot visualization for “agonist fingerprinting” of β-AR receptor expression using the Δlog(E_max_/EC_50_) parameter, and we provide real-world examples of the utility of Δlog(E_max_/EC_50_) in assessing new cell systems and measuring potential ligand bias. This work builds on that of others (Winpenny et al., 2016; Kenakin, 2017), particularly where similar representations have been used to visualize receptor selectivity in tissues (Kenakin, 2017) or ligand bias (Herenbrink et al., 2016). Because the Δlog(E_max_/EC_50_) value for a given test agonist–reference agonist pair is a system-independent measure of agonism, regardless of factors such as receptor expression level or signal transduction efficiency, this method provides a functional readout advantage over profiling receptor expression with selective antagonists.

The use of multiple agonists for profiling lessens the chance that the expression of a closely related receptor is missed due to the use of non-selective ligands. However, it is worth noting that potentially only one test agonist and a single reference agonist may be required for Δlog(E_max_/EC_50_) to be effective in identifying receptor expression, as long as the agonist pair shows a relationship specific to the receptor of interest. For example, the isoprenaline-referenced Δlog(E_max_/EC_50_) value of formoterol is significantly different for β_1_-AR (−0.87 ± 0.15), β_2_-AR (1.17 ± 0.15), and β_3_-AR (−0.02 ± 0.1). To test a novel system, a dose-response of isoprenaline and formoterol and a formoterol Δlog(E_max_/EC_50_) calculation should allow assessment of β-AR subtype expression at a population level, agnostic of the expression level or coupling efficiency of that system. This finding extends the utility of the approach to other receptor types with a limited number of known agonists. Applying agonist fingerprinting to novel systems will be most successful when the assay format allows direct comparison of test and reference agonist (i.e., plate-matching, as in our study) to minimize replicate error in Δlog(E_max_/EC_50_) measurements. To provide an example of measurement error if plate-matching is not possible, we have provided a further analysis of a data subset in Supplementary Table S3.

We observed unique Δlog(E_max_/EC_50_) agonist fingerprints when CHO-K1 cells with single β-AR expression were mixed in different proportions, and the fingerprint changed in a predictable continuum between the pure receptor populations based on those receptor subtype proportions. In addition, we found that rat primary cortical astrocytes, C6 rat glioma cells, and human THP-1 cells naturally express both β_1_-AR and β_2_-AR, examples of the endogenous expression of multiple β-ARs. The Δlog(E_max_/EC_50_) agonist fingerprint can detect these co-expression scenarios and can even provide clues about the relative expression of receptor subtypes, though this can be difficult to detect when one subtype is expressed more than the other (Figures 2, 3). For example, THP-1 cells naturally express a small population of functional β_2_-AR (relative to β_1_-AR), which is less obvious in the Δlog(E_max_/EC_50_) β-AR agonist fingerprints. Thus, it is essential to carefully examine the dose–response curves in all situations; a shallow Hill slope from biphasic dose–response curves with selective agonists (such as formoterol for THP-1 cells) was the biggest indicator of potential dual expression.

Expanding Δlog(E_max_/EC_50_) calculations to additional signaling pathways, we show that for β_2_-AR, the Δlog(E_max_/EC_50_) value for cAMP production tracks closely with the Δlog(E_max_/EC_50_) value obtained from β-arrestin recruitment and functional desensitization assays across multiple agonists, assayed with PathHunter^®^ and 1321N1 cells, respectively. With the exception of formoterol and levosalbutamol compared in cAMP versus functional desensitization assays, ΔΔlog(E_max_/EC_50_) values include the value of 0, indicating a lack of bias (Supplementary Table S4). This includes isoetharine, previously reported as β-arrestin-biased (Drake et al., 2008; Ippolito and Benovic, 2021). Our data suggest that at the cellular level, the β_2_-AR agonists tested here do not display ligand bias for cAMP signaling or β-arrestin recruitment. This is in line with previous studies, which have noted that different assessments of β_2_-AR agonist bias can be derived from different quantification methods and that the magnitude of β_2_-AR bias is small (Rajagopal et al., 2011; Onaran et al., 2017) compared to that of other receptors (Winpenny et al., 2016; Onaran et al., 2017).

Our results suggest that meaningful ligand bias is not present among our tested β_2_-AR agonists for the functional effects we measured (cAMP, β-arrestin recruitment, and functional desensitization), but this finding may not be generalizable to all tissue types expressing β_2_-AR. A major challenge in translating ligand bias to a therapeutic effect is to understand what magnitude of ligand bias in an *in vitro* system corresponds to a clinically meaningful pharmacodynamic effect. Without a clearly biased ligand, this cannot be tested effectively. Therapeutically selective activation of pathways may still be attained by different molecular and therapeutic properties not studied here, namely, lipophilicity, occupancy at equi-effective concentration, and dosing schedule (Düringer et al., 2009). A key takeaway message from our studies and those of others (Düringer et al., 2009) is that, with sustained treatment, unbiased partial agonists desensitize partially. The sensitivity of the target tissue, dosing regimen, and system re-sensitization will determine both the degree of signaling stimulus imparted and its potential for attenuation in therapeutic programs.

In the brain, noradrenaline is released broadly in the cortex and cerebellum in a manner reflecting a ‘volume transmission’ process, from varicosities on neuronal noradrenergic axons with cell bodies originating mainly in the locus coeruleus (Schwarz and Luo, 2015). Using agonist fingerprinting, we demonstrate β_2_-AR expression in human primary pericytes, endothelial cells, vascular smooth muscle cells, cerebral astrocytes, and hippocampal astrocytes. In precursor cell systems, we show β_2_-AR expression in iPSC microglia and cortex-derived neural progenitor cells. The observed expression of β_2_-AR on several human non-neuronal cell types provides support for this receptor and these cell types as being recipients of noradrenaline volume transmission, in line with observations of multi-cellular expression of β-ARs in the human brain (Shimohama et al., 1987; Kalaria and Harik, 1989; Mantyh et al., 1995; Tsukahara et al., 2018). Our observations that rodent systems show broad dual β_1_-AR/β_2_-AR expression are also consistent with those of prior tissue analyses (Rainbow et al., 1984; Shao and Sutin, 1992; Asashima et al., 2003). Several groups have mechanistically linked β-ARs with CNS physiology and homeostasis (Gibbs et al., 2009; Catus et al., 2011; Hertz et al., 2013; Coutellier et al., 2014; O’Dell et al., 2015; Hagena et al., 2016; Ardestani et al., 2017), setting the stage for brain active β-AR therapeutics for indications that feature early impairment of locus coeruleus function. Our results show a similar potency for isoprenaline across most cell systems, which may provide a useful translational starting point for achieving a particular pharmacologic response. Successful translation will also require innovative ways to selectively deliver or activate central versus peripheral receptors to achieve target tissue specificity.

## Data Availability

The original contributions presented in the study are included in the article/Supplementary Material; further inquiries can be directed to the corresponding author.
