# Supplementary material for: Fingerprinting heterocellular β-adrenoceptor functional expression in the brain using agonist activity profiles
Source: Front Mol Biosci. 2023 Aug 17;10:1214102. doi: 10.3389/fmolb.2023.1214102 (PMC10471193; doi:10.3389/fmolb.2023.1214102)
Supplement: Supplementary file 1 [file DataSheet1.PDF]

Supplementary Material

Fingerprinting heterocellular β-adrenergic receptor functional expression in the brain using agonist activity profiles

Rachel A Matt.†*, Frederick G. Westhorpe†, Rosemary F. Romuar, Payal Rana, Joel R. Gever, Anthony P. Ford

*Correspondence: matt@curasen.com

1 Supplementary Figures and Tables

1.1 Supplementary Table 1: Cell sources, cell line engineering and culture conditions

Name	Description	Source	Exogenous protein Accession #	Growth media	Media, growth factor sources
CHO-K1	Chinese Hamster Ovary	ATCC CCL-61	NA	DMEM/F:12, 10% FBS, 1% P/S	Caisson DFL13
CHO-K1 hβ_1-AR	Chinese Hamster Ovary – exogenous hβ_1-AR	Eurofins DiscoverX 95-0113C2	NP_000675	DMEM/F:12, 10% FBS, 1% P/S	Caisson DFL13
CHO-K1 hβ_2-AR	Chinese Hamster Ovary – exogenous hβ_2-AR	Eurofins DiscoverX 95-0121C2	NP_000015	DMEM/F:12, 10% FBS, 1% P/S	Caisson DFL13
CHO-K1 hβ_3-AR	Chinese Hamster Ovary – exogenous hβ_3-AR	CuraSen	NP_000016	DMEM/F:12, 10% FBS, 1% P/S	Caisson DFL13
ReNcell VM (-/+ hβ2-AR)	Human immortalized neural progenitor cell, ventral mesencephalon	EMD Millipore SCC008	NP_000015	Vendor supplied media	EMD Millipore SCM005
ReNcell CX	Human immortalized neural progenitor cell, cerebral cortex	EMD Millipore SCC007	NA	Vendor supplied media	EMD Millipore SCM005

Name	Description	Source	Exogenous protein Accession #	Growth media	Media, growth factor sources
THP-1	Human monocyte	ATCC TIB-202	NA	RPMI-1640, 10% FBS, 1% P/S	Corning 10-104-CV
C6	Rat glioma	ATCC CCL-107	NA	F-12 K, 10% FBS, 1% P/S	Corning 10-025-CV
1321N1	Human astrocytoma	Sigma 86030402	NA	MEM, 10% FBS, 1% P/S	Corning 10-010-CV
HMC3	Human immortalized microglial cell	ATCC CRL-3304	NA	MEM, 10% FBS, 1% P/S	Corning 10-010-CV
HBEC-5i	Human immortalized cerebral microvascular endothelial cell	ATCC CRL-3245	NA	DMEM:F-12 10% FBS, 1% P/S, containing 40 ug/ml ECGS	Caisson DFL13, Corning 354006
Human cerebellar astrocytes	Human primary fetal cerebellar astrocytes	Sciencell #1810 Lot 22351	NA	Vendor supplied media	Sciencell #1801
Human hippocampal astrocytes	Human primary fetal hippocampal astrocytes	Sciencell #1830 Lot 17337	NA	Vendor supplied media	Sciencell #1801
Human pericytes (Neuromics)	Human brain primary microvascular pericytes	Neuromics HMP104	NA	Vendor supplied media	Neuromics PGB001
Human pericytes (Sciencell)	Human brain primary fetal vascular pericytes	Sciencell #1200	NA	Vendor supplied media	Sciencell #1201
Human brain vascular smooth muscle cells	Human brain primary fetal vascular smooth muscle cells	Sciencell #1100 Lot 27218	NA	Vendor supplied media	Sciencell #1101
iMicroglia	Human iPSC-derived microglia	Tempo Biosciences iMG	NA	iMG media	Tempo Biosciences

1.2 **Supplementary Table 2:** Summary pharmacology data, plate-matched Δlog(Emax/EC50)

	CHO-K1 recombinant β_1-AR							
	Mean pEC50	pEC50 (SD)	Mean Emax	Emax (SD)	Mean Hill slope	Mean Isoprenaline Δlog(Emax/EC50)	95% CI Isoprenaline Δlog(Emax/EC50)	n
Isoprenaline	9.65	0.32	100	0	0.84	0	0	21
Adrenaline	8.44	0.28	101	1	0.78	-1.24	0.11	12
Noradrenaline	8.6	0.28	101	1	0.83	-1.06	0.09	18
Dobutamine	8.02	0.24	99	1	0.83	-1.67	0.07	14
Prenalterol	8.56	0.18	82	8	0.95	-1.25	0.08	9
Formoterol	8.91	0.47	99	2	0.84	-0.87	0.15	11
Clenbuterol	7.89	0.23	76	5	0.91	-1.86	0.04	16
Salbutamol	6.61	0.18	89	4	0.96	-3.16	0.09	11
Tulobuterol	6.9	0.23	74	9	0.9	-2.74	0.12	10
Mirabegron	7.02	0.25	91	4	0.95	-2.95	0.15	4

	CHO-K1 recombinant β_2-AR							
	Mean pEC50	pEC50 (SD)	Mean Emax	Emax (SD)	Mean Hill slope	Mean Isoprenaline Δlog(Emax/EC50)	95% CI Isoprenaline Δlog(Emax/EC50)	n
Isoprenaline	9.24	0.3	100	0	0.88	0	0	22
Adrenaline	8.89	0.3	101	1	0.86	-0.36	0.07	13
Noradrenaline	6.96	0.25	100	2	0.93	-2.3	0.09	19
Dobutamine	7.16	0.12	80	7	0.93	-2.26	0.08	15
Prenalterol	7.4	0.16	30	11	1.23	-2.39	0.07	9
Formoterol	10.51	0.34	100	1	0.89	1.17	0.15	11
Clenbuterol	9.28	0.24	91	3	0.91	0.03	0.06	17
Salbutamol	7.98	0.26	96	2	0.91	-1.37	0.09	12
Tulobuterol	7.91	0.33	76	8	0.88	-1.28	0.14	10
Mirabegron	6.56	0.25	68	15	1.17	-3.21	0.2	5

	CHO-K1 recombinant β_3-AR							
	Mean pEC50	pEC50 (SD)	Mean Emax	Emax (SD)	Mean Hill slope	Mean Isoprenaline Δlog(Emax/EC50)	95% CI Isoprenaline Δlog(Emax/EC50)	n
Isoprenaline	8.36	0.14	100	0	0.87	0	0	23
Adrenaline	7.52	0.21	100	1	0.85	-0.86	0.12	4
Noradrenaline	7.66	0.16	100	2	0.88	-0.68	0.07	5
Dobutamine	6.88	0.12	99	1	0.89	-1.44	0.07	4
Prenalterol	6.34	0.22	87	4	0.77	-2.13	0.18	3
Formoterol	8.31	0.22	100	3	0.86	-0.02	0.1	8
Clenbuterol	6.44	0.24	84	5	1.02	-2.13	0.17	6
Salbutamol	6.03	0.15	95	2	1.04	-2.32	0.11	7
Tulobuterol	6.02	0.03	90	1	0.99	-2.58	0.06	3
Mirabegron	9.42	0.16	93	3	0.7	1.09	0.13	6

1.2 Supplementary Table 2, continued: Summary pharmacology data, plate-matched Δlog(Emax/EC50)

	C6 Parent							
	Mean pEC50	pEC50 (SD)	Mean Emax	Emax (SD)	Mean Hill slope	Mean Isoprenaline Δlog(Emax/EC50)	95% CI Isoprenaline Δlog(Emax/EC50)	n
Isoprenaline	9.29	0.24	100	0	1	0	0	10
Adrenaline	8.06	0.19	98	2	1.11	-1.21	0.06	5
Noradrenaline	7.98	0.43	98	2	1.07	-1.27	0.17	4
Dobutamine	7.74	0.18	99	1	1.11	-1.72	0.09	3
Prenalterol	8.23	0.15	81	6	0.9	-1.32	0.04	3
Formoterol	8.56	0.2	99	3	0.68	-0.4	0.21	3
Clenbuterol	8	0.14	79	1	0.89	-1.55	0.08	3
Salbutamol	6.53	0.28	90	8	0.82	-2.77	0.19	5
Tulobuterol	6.72	0.35	65	18	0.85	-2.8	0.22	5

	C6 Rat Glioma: Adrb1 KO							
	Mean pEC50	pEC50 (SD)	Mean Emax	Emax (SD)	Mean Hill slope	Mean Isoprenaline Δlog(Emax/EC50)	95% CI Isoprenaline Δlog(Emax/EC50)	n
Isoprenaline	8.22	0.18	100	0	0.9	0	0	22
Adrenaline	7.44	0.15	93	4	0.96	-0.8	0.09	9
Noradrenaline	6.06	0.22	86	5	0.89	-2.22	0.17	8
Dobutamine	6.08	0.18	67	5	0.88	-2.35	0.14	6
Prenalterol	ND	NA	>20	NA	NA	NA	NA	2
Formoterol	9.4	0.33	92	1	0.71	1.18	0.24	5
Clenbuterol	7.94	0.18	64	8	0.85	-0.48	0.14	9
Salbutamol	6.5	0.34	78	4	0.86	-1.74	0.25	5
Tulobuterol	6.82	0.19	42	7	0.86	-1.77	0.15	6

	C6 Rat Glioma: Adrb2 KO							
	Mean pEC50	pEC50 (SD)	Mean Emax	Emax (SD)	Mean Hill slope	Mean Isoprenaline Δlog(Emax/EC50)	95% CI Isoprenaline Δlog(Emax/EC50)	n
Isoprenaline	8.87	0.28	100	0	1.07	0	0	91
Adrenaline	7.54	0.23	97	4	1.04	-1.3	0.13	16
Noradrenaline	7.28	0.45	99	2	1.1	-1.54	0.24	15
Dobutamine	7	0.29	96	3	1.05	-1.94	0.12	10
Prenalterol	7.85	0.25	56	9	1.04	-1.39	0.28	5
Formoterol	8.19	0.27	98	2	1.01	-0.83	0.13	8
Clenbuterol	7.1	0.21	44	11	0.84	-2.15	0.12	26
Salbutamol	5.87	0.11	82	4	0.95	-3.17	0.22	11
Tulobuterol	6.31	0.17	43	13	0.93	-2.95	0.13	23

1.2 Supplementary Table 2, continued: Summary pharmacology data, plate-matched Δlog(Emax/EC50)

	Rat Primary Astrocytes							
	Mean pEC50	pEC50 (SD)	Mean Emax	Emax (SD)	Mean Hill slope	Mean Isoprenaline Δlog(Emax/EC50)	95% CI Isoprenaline Δlog(Emax/EC50)	n
Isoprenaline	8.71	0.31	100	0	1.11	0	0	10
Adrenaline	7.54	0.31	98	2	1.02	-1.15	0.25	4
Noradrenaline	7.12	0.33	99	2	1.08	-1.54	0.31	5
Dobutamine	7.06	0.29	95	5	1.08	-1.69	0.06	6
Prenalterol	7.71	0.4	45	18	1.2	-1.41	0.31	6
Formoterol	8.79	0.3	102	4	0.63	-0.07	0.31	4
Clenbuterol	7.88	0.27	70	10	0.96	-1.02	0.32	5
Salbutamol	6.4	0.33	84	3	0.85	-2.45	0.4	4
Tulobuterol	6.51	0.19	47	10	0.95	-2.44	0.31	4
	**Human Brain Endothelial Cell (HBEC-5i)**							
	Mean pEC50	pEC50 (SD)	Mean Emax	Emax (SD)	Mean Hill slope	Mean Isoprenaline Δlog(Emax/EC50)	95% CI Isoprenaline Δlog(Emax/EC50)	n
Isoprenaline	8.09	0.1	100	0	0.99	0	0	8
Adrenaline	7.77	0.19	95	2	1.07	-0.35	0.09	4
Noradrenaline	5.32	0.02	100	5	0.96	-2.87	0.19	4
Dobutamine	5.9	0.12	29	3	0.77	-2.74	0.11	4
Prenalterol	ND	NA	<5	NA	NA	NA	NA	4
Formoterol	9.68	0.13	98	1	1.16	1.58	0.04	4
Clenbuterol	8.54	0.12	67	6	1.11	0.27	0.06	4
Salbutamol	6.88	0.08	81	4	1.08	-1.3	0.12	4
Tulobuterol	7.26	0.11	39	6	1.09	-1.25	0.12	4
	**Primary Human Pericytes (Neuromics)**							
	Mean pEC50	pEC50 (SD)	Mean Emax	Emax (SD)	Mean Hill slope	Mean Isoprenaline Δlog(Emax/EC50)	95% CI Isoprenaline Δlog(Emax/EC50)	n
Isoprenaline	8.88	0.32	100	0	1.43	0	0	8
Adrenaline	8.32	0.41	100	2	1.42	-0.58	0.12	4
Noradrenaline	6.44	0.17	104	3	1.21	-2.45	0.31	4
Dobutamine	6.44	0.24	83	15	1.11	-2.51	0.15	4
Prenalterol	6.56	0.28	15	8	1.08	-3.29	0.33	4
Formoterol	10.33	0.32	93	3	1.4	1.4	0.12	4
Clenbuterol	8.91	0.35	97	4	1.14	-0.01	0.07	4
Salbutamol	8.03	0.36	101	2	1.16	-0.87	0.17	4
Tulobuterol	7.58	0.12	70	18	1.12	-1.44	0.21	4

1.2 Supplementary Table 2, continued: Summary pharmacology data, plate-matched Δlog(Emax/EC50)

	iPSC Microglia (Tempo Bio)							
	Mean pEC50	pEC50 (SD)	Mean Emax	Emax (SD)	Mean Hill slope	Mean Isoprenaline Δlog(Emax/EC50)	95% CI Isoprenaline Δlog(Emax/EC50)	n
Isoprenaline	8.19	0.14	100	0	1.03	0	0	8
Adrenaline	7.79	0.13	98	2	0.88	-0.41	0.13	4
Noradrenaline	5.93	0.15	104	12	0.77	-2.25	0.26	4
Dobutamine	6.66	0.25	34	7	1.03	-2	0.2	4
Prenalterol	ND	NA	<5	NA	NA	NA	NA	4
Formoterol	9.14	0.18	79	5	1.09	0.85	0.14	4
Clenbuterol	8.53	0.16	52	6	1.15	0.06	0.13	4
Salbutamol	7.04	0.11	76	6	0.94	-1.27	0.13	4
Tulobuterol	7.35	0.16	34	8	1.05	-1.31	0.15	4
	**Human Cortex Neural Progenitor Cells (ReNcell CX)**							
	Mean pEC50	pEC50 (SD)	Mean Emax	Emax (SD)	Mean Hill slope	Mean Isoprenaline Δlog(Emax/EC50)	95% CI Isoprenaline Δlog(Emax/EC50)	n
Isoprenaline	8.91	0.15	100	0	1.05	0	0	9
Adrenaline	8.69	0.27	99	2	1.07	-0.32	0.12	4
Noradrenaline	6.15	0.23	97	3	1.06	-2.79	0.16	7
Dobutamine	6.42	0.1	61	6	1.01	-2.7	0.14	6
Prenalterol	6.78	0.22	7	1	0.82	-3.21	0.06	3
Formoterol	10.25	0.06	100	1	1.14	1.42	0.11	6
Clenbuterol	8.95	0.21	90	3	0.99	-0.09	0.14	4
Salbutamol	7.61	0.22	94	3	1.18	-1.31	0.13	6
Tulobuterol	7.58	0.08	75	4	1.13	-1.3	0.2	2
	**Human Ventral Mesencephalon Neural Progenitor cells (ReNcell VM): ADRB2 transgene**							
	Mean pEC50	pEC50 (SD)	Mean Emax	Emax (SD)	Mean Hill slope	Mean Isoprenaline Δlog(Emax/EC50)	95% CI Isoprenaline Δlog(Emax/EC50)	n
Isoprenaline	9.96	0.4	100	0	0.92	0	0	12
Adrenaline	9.54	0.33	98	2	0.92	-0.63	0.22	6
Noradrenaline	7.51	0.5	99	3	0.84	-2.65	0.09	6
Dobutamine	7.19	0.13	96	6	0.86	-2.61	0.11	6
Prenalterol	7.55	0.11	52	7	0.94	-2.51	0.09	6
Formoterol	11.3	0.18	111	1	0.88	1.51	0.27	4
Clenbuterol	10.07	0.52	99	2	0.9	0.02	0.09	6
Salbutamol	8.55	0.27	103	5	0.89	-1.21	0.18	6
Tulobuterol	8.74	0.45	100	5	0.93	-1.28	0.1	6

1.2 Supplementary Table 2, continued: Summary pharmacology data, plate-matched Δlog(Emax/EC50)

	Primary Human Pericytes (Sciencell)							
	Mean pEC50	pEC50 (SD)	Mean Emax	Emax (SD)	Mean Hill slope	Mean Isoprenaline Δlog(Emax/EC50)	95% CI Isoprenaline Δlog(Emax/EC50)	n
Isoprenaline	8.38	0.3	100	0	0.93	0	0	8
Adrenaline	7.98	0.39	100	1	0.92	-0.38	0.1	4
Noradrenaline	5.82	0.27	101	6	0.89	-2.54	0.06	4
Dobutamine	6.45	0.17	35	15	0.84	-2.45	0.09	4
Prenalterol	6.72	0.24	6	1	1.09	-3.18	0.04	4
Formoterol	9.74	0.2	96	2	1.02	1.37	0.2	4
Clenbuterol	8.72	0.17	69	18	1	0.18	0.04	4
Salbutamol	7.16	0.29	81	12	0.97	-1.29	0.07	4
Tulobuterol	7.44	0.39	48	18	1.32	-1.32	0.24	4
	Human Cerebellar Astrocytes (Sciencell)							
	Mean pEC50	pEC50 (SD)	Mean Emax	Emax (SD)	Mean Hill slope	Mean Isoprenaline Δlog(Emax/EC50)	95% CI Isoprenaline Δlog(Emax/EC50)	n
Isoprenaline	8.66	0.09	100	0	1.07	0	0	8
Adrenaline	8.27	0.09	100	2	1.04	-0.37	0.09	4
Noradrenaline	6.08	0.19	99	3	1.01	-2.56	0.08	4
Dobutamine	6.49	0.12	42	7	0.97	-2.56	0.12	4
Prenalterol	ND	NA	<5	NA	NA	NA	NA	4
Formoterol	9.96	0.3	99	1	1.14	1.31	0.21	4
Clenbuterol	8.8	0.09	82	3	1.12	0.07	0.06	4
Salbutamol	7.39	0.07	91	3	1.09	-1.29	0.05	4
Tulobuterol	7.53	0.19	59	7	1.06	-1.38	0.21	4
	Human Hippocampal Astrocytes (Sciencell)							
	Mean pEC50	pEC50 (SD)	Mean Emax	Emax (SD)	Mean Hill slope	Mean Isoprenaline Δlog(Emax/EC50)	95% CI Isoprenaline Δlog(Emax/EC50)	n
Isoprenaline	8.47	0.26	100	0	0.96	0	0	19
Adrenaline	8.21	0.27	100	2	0.96	-0.25	0.07	8
Noradrenaline	5.79	0.46	100	2	0.93	-2.85	0.36	9
Dobutamine	6.12	0.1	37	13	0.89	-2.77	0.14	6
Prenalterol	ND	NA	<5	NA	NA	NA	NA	4
Formoterol	9.92	0.17	99	1	0.98	1.61	0.16	4
Clenbuterol	8.71	0.09	84	3	0.96	0	0.11	6
Salbutamol	7.34	0.17	89	3	1.07	-1.39	0.04	4
Tulobuterol	7.28	0.12	51	10	0.98	-1.43	0.05	7

1.2 Supplementary Table 2, continued: Summary pharmacology data, plate-matched Δlog(Emax/EC50)

	Human Brain Vascular Smooth Muscle Cells (Sciencell)							
	Mean pEC50	pEC50 (SD)	Mean Emax	Emax (SD)	Mean Hill slope	Mean Isoprenaline Δlog(Emax/EC50)	95% CI Isoprenaline Δlog(Emax/EC50)	n
Isoprenaline	8.25	0.09	100	0	1.04	0	0	8
Adrenaline	7.84	0.08	102	2	0.95	-0.42	0.1	4
Noradrenaline	6.03	0.51	104	11	0.86	-2.21	0.37	4
Dobutamine	6.32	0.08	40	7	1.04	-2.33	0.08	4
Prenalterol	ND	NA	<5	NA	NA	NA	NA	4
Formoterol	9.83	0.17	98	2	1.07	1.56	0.09	4
Clenbuterol	8.59	0.08	70	8	1.09	0.17	0.06	4
Salbutamol	7.08	0.18	83	6	1.02	-1.26	0.12	4
Tulobuterol	7.3	0.16	45	6	1.05	-1.3	0.17	4
	**Human Microglia Cell Line (HMC3)**							
	Mean pEC50	pEC50 (SD)	Mean Emax	Emax (SD)	Mean Hill slope	Mean Isoprenaline Δlog(Emax/EC50)	95% CI Isoprenaline Δlog(Emax/EC50)	n
Isoprenaline	8.07	0.17	100	0	1.08	0	0	8
Adrenaline	7.56	0.14	89	5	1.04	-0.55	0.18	4
Noradrenaline	5.75	0.22	99	12	0.76	-2.32	0.13	4
Dobutamine	6.31	0.24	32	8	0.86	-2.28	0.26	4
Prenalterol	ND	NA	<5	NA	NA	NA	NA	4
Formoterol	9.1	0.15	82	6	1.27	0.95	0.1	4
Clenbuterol	8.31	0.15	45	12	1.14	-0.12	0.07	4
Salbutamol	6.88	0.13	65	9	1.1	-1.38	0.08	4
Tulobuterol	7.09	0.08	23	8	0.9	-1.65	0.06	4
	**THP-1 Human Monocyte**							
	Mean pEC50	pEC50 (SD)	Mean Emax	Emax (SD)	Mean Hill slope	Mean Isoprenaline Δlog(Emax/EC50)	95% CI Isoprenaline Δlog(Emax/EC50)	n
Isoprenaline	8.67	0.27	100	0	1.2	0	0	14
Adrenaline	7.52	0.31	101	1	1.06	-1.11	0.06	7
Noradrenaline	7.55	0.27	100	1	1.21	-1.08	0.06	7
Dobutamine	7.05	0.27	93	5	1.21	-1.62	0.05	7
Prenalterol	7.34	0.36	31	10	0.87	-1.82	0.22	7
Formoterol	7.77	0.42	98	4	0.73	-0.87	0.15	7
Clenbuterol	6.87	0.3	56	7	0.51	-2.16	0.23	7
Salbutamol	5.71	0.37	73	10	0.81	-3.1	0.21	5
Tulobuterol	6	0.15	46	16	0.59	-3.02	0.13	5

1.2 Supplementary Table 2, continued: Summary pharmacology data, plate-matched Δlog(Emax/EC50)

	THP-1 Human Monocyte: ADRB1 KO							
	Mean pEC50	pEC50 (SD)	Mean Emax	Emax (SD)	Mean Hill slope	Mean Isoprenaline Δlog(Emax/EC50)	95% CI Isoprenaline Δlog(Emax/EC50)	n
Isoprenaline	7.85	0.12	100	0	0.88	0	0	13
Adrenaline	7.45	0.17	101	8	0.84	-0.44	0.14	6
Noradrenaline	5.27	0.13	82	10	0.94	-2.58	0.04	6
Dobutamine	ND	NA	>20	NA	NA	NA	NA	6
Prenalterol	ND	NA	<5	NA	NA	NA	NA	6
Formoterol	8.91	0.19	86	8	0.98	0.96	0.17	6
Clenbuterol	8.28	0.27	39	6	0.88	-0.01	0.18	6
Salbutamol	6.39	0.35	55	10	0.62	-1.71	0.21	5
Tulobuterol	ND	NA	>30	NA	NA	NA	NA	5

	THP-1 Human Monocyte: ADRB2 KO							
	Mean pEC50	pEC50 (SD)	Mean Emax	Emax (SD)	Mean Hill slope	Mean Isoprenaline Δlog(Emax/EC50)	95% CI Isoprenaline Δlog(Emax/EC50)	n
Isoprenaline	8.7	0.2	100	0	1.15	0	0	13
Adrenaline	7.53	0.21	100	0	1.1	-1.15	0.03	6
Noradrenaline	7.66	0.16	100	0	1.17	-1.03	0.06	6
Dobutamine	7.1	0.21	95	3	1.14	-1.59	0.05	6
Prenalterol	7.5	0.14	35	8	0.99	-1.63	0.07	6
Formoterol	7.52	0.22	97	3	1.04	-1.18	0.06	6
Clenbuterol	6.33	0.2	53	3	0.53	-2.72	0.17	6
Salbutamol	5.51	0.12	67	11	0.97	-3.33	0.06	5
Tulobuterol	5.92	0.16	41	3	0.78	-3.18	0.08	5

	CHO-K1: 75% β_1-AR, 25% β_2-AR							
	Mean pEC50	pEC50 (SD)	Mean Emax	Emax (SD)	Mean Hill slope	Mean Isoprenaline Δlog(Emax/EC50)	95% CI Isoprenaline Δlog(Emax/EC50)	n
Isoprenaline	9.53	0.26	100	0	0.84	0	0	10
Adrenaline	8.6	0.15	100.77	0.6	0.85	-0.95	0.2	5
Noradrenaline	8.47	0.29	100.38	0.63	0.74	-1.05	0.1	10
Dobutamine	7.83	0.19	95.39	1.39	0.82	-1.68	0.1	9
Prenalterol	8.38	0.16	71.83	6.28	0.94	-1.31	0.09	6
Formoterol	9.59	0.39	97.12	2.28	0.75	0.02	0.17	5
Clenbuterol	8.62	0.23	80.18	3.29	0.77	-1.01	0.09	10
Salbutamol	7.15	0.09	91.08	3.96	0.76	-2.44	0.2	5
Tulobuterol	7.4	0.11	78.05	5.43	0.8	-2.21	0.15	5

1.2 Supplementary Table 2, continued: Summary pharmacology data, plate-matched Δlog(Emax/EC50)

	Mean pEC50	pEC50 (SD)	Mean Emax	Emax (SD)	Mean Hill slope	Mean Isoprenaline Δlog(Emax/EC50)	95% CI Isoprenaline Δlog(Emax/EC50)	n
**CHO-K1: 50% β_1-AR, 50% β_2-AR**								
Isoprenaline	9.44	0.22	100	0	0.82	0	0	11
Adrenaline	8.7	0.09	101.09	0.93	0.9	-0.78	0.16	6
Noradrenaline	8.18	0.28	99.84	1.32	0.69	-1.26	0.12	11
Dobutamine	7.66	0.17	91.51	1.37	0.78	-1.8	0.1	10
Prenalterol	8.19	0.16	65.2	7.75	0.95	-1.45	0.11	6
Formoterol	9.98	0.28	98.18	1.06	0.8	0.49	0.13	6
Clenbuterol	8.92	0.17	85.23	2.91	0.82	-0.59	0.1	11
Salbutamol	7.57	0.12	92.36	2.02	0.82	-1.95	0.16	6
Tulobuterol	7.75	0.07	77.31	4.54	0.82	-1.76	0.16	5
**CHO-K1: 25% β_1-AR, 75% β_2-AR**								
	Mean pEC50	pEC50 (SD)	Mean Emax	Emax (SD)	Mean Hill slope	Mean Isoprenaline Δlog(Emax/EC50)	95% CI Isoprenaline Δlog(Emax/EC50)	n
Isoprenaline	9.34	0.22	100	0	0.83	0	0	11
Adrenaline	8.79	0.11	101.1	0.72	0.86	-0.58	0.17	6
Noradrenaline	7.74	0.27	100.26	0.93	0.66	-1.6	0.11	11
Dobutamine	7.46	0.15	86.54	3.42	0.78	-1.93	0.1	10
Prenalterol	8	0.2	51.67	7.7	1.06	-1.63	0.2	6
Formoterol	10.19	0.26	98.96	0.69	0.86	0.81	0.17	6
Clenbuterol	9.16	0.14	87.8	2.25	0.87	-0.24	0.07	11
Salbutamol	7.75	0.15	93.54	1.89	0.9	-1.66	0.19	6
Tulobuterol	7.93	0.11	79.33	4.69	0.8	-1.48	0.16	5
**1321N1**								
	Mean pEC50	pEC50 (SD)	Mean Emax	Emax (SD)	Mean Hill slope	Mean Isoprenaline Δlog(Emax/EC50)	95% CI Isoprenaline Δlog(Emax/EC50)	n
Isoprenaline	8.49	0.22	1	0	1.12	0	0	141
Adrenaline	7.98	0.19	1.01	0.01	1.07	-0.25	0.27	7
Noradrenaline	5.78	0.21	1.01	0.02	1.02	-2.45	0.39	8
Dobutamine	6.3	0.29	0.45	0.08	0.98	-2.55	0.24	5
Prenalterol	ND	NA	<0.10	NA	NA	NA	NA	3
Formoterol	9.96	0.18	0.99	0	1.11	1.44	0.17	5
Clenbuterol	8.61	0.26	0.84	0.05	1.11	0.02	0.13	22
Salbutamol	7.25	0.2	0.92	0.02	1.06	-1.24	0.21	9
Tulobuterol	7.2	0.23	0.58	0.11	0.96	-1.42	0.18	20
Terbutaline	7.08	0.39	0.91	0.03	1.11	-1.55	0.31	6
Levosalbutamol	7.4	0.22	0.89	0.04	1.04	-1.09	0.15	5
Isoetharine	7.58	0.13	0.99	0.01	0.97	-1.05	0.19	5

1.2 Supplementary Table 2, continued: Summary pharmacology data, plate-matched Δlog(Emax/EC50)

	1321N1 - cAMP standard curve normalized data							
	Mean pEC50	pEC50 (SD)	Mean Emax	Emax (SD)	Mean Hill slope	Mean Isoprenaline Δlog(Emax/EC50)	95% CI Isoprenaline Δlog(Emax/EC50)	n
Isoprenaline	7.45	0.21	1	0	1.12	0		141
Adrenaline	6.96	0.18	1.06	0.07	1.28	-0.22	0.22	7
Noradrenaline	<5.3	ND	<0.9	ND	ND	ND	ND	8
Dobutamine	6.03	0.34	0.06	0.01	1.07	-2.82	0.2	5
Prenalterol	NA	ND	<0.05	ND	ND	ND	ND	4
Formoterol	8.94	0.27	0.92	0.06	1.2	1.42	0.1	5
Clenbuterol	7.96	0.2	0.25	0.04	1.14	-0.05	0.05	22
Salbutamol	6.45	0.15	0.44	0.04	1.05	-1.32	0.22	9
Tulobuterol	6.8	0.2	0.09	0.03	1.04	-1.61	0.24	20

	DiscoverX PathHunter β_2-AR - cAMP							
	Mean pEC50	pEC50 (SD)	Mean Emax	Emax (SD)	Mean Hill slope	Mean Isoprenaline Δlog(Emax/EC50)	95% CI Isoprenaline Δlog(Emax/EC50)	n
Isoprenaline	10.19	0.18	1.00	0.00	1.49	0	0	13
Adrenaline	9.87	0.26	1.02	0.03	1.37	-0.26	0.07	6
Noradrenaline	7.63	0.11	0.96	0.05	1.45	-2.56	0.07	5
Prenalterol	7.49	0.12	0.58	0.10	1.03	-2.96	0.11	4
Formoterol	11.43	0.69	0.97	0.04	1.43	1.17	0.5	5
Clenbuterol	10.21	0.2	0.97	0.04	1.56	0.02	0.04	5
Salbutamol	8.75	0.17	0.98	0.02	1.4	-1.43	0.05	5
Levosalbutamol	9.19	0.26	0.98	0.02	1.46	-1.06	0.14	4
Tulobuterol	9.00	0.14	0.98	0.03	1.7	-1.33	0.04	4
Terbutaline	8.31	0.18	1.01	0.01	1.49	-2.00	0.09	4
Isoetharine	9.12	0.1	1.05	0.04	1.24	-1.07	0.21	4

	DiscoverX PathHunter β_2-AR - Arrestin Recruitment							
	Mean pEC50	pEC50 (SD)	Mean Emax	Emax (SD)	Mean Hill slope	Mean Isoprenaline Δlog(Emax/EC50)	95% CI Isoprenaline Δlog(Emax/EC50)	n
Isoprenaline	7.34	0.16	1.00	0.00	1.17	0	0	12
Adrenaline	6.84	0.32	0.99	0.03	1.04	-0.46	0.12	3
Noradrenaline	4.81	0.05	0.85	0.09	1.14	-2.58	0.19	3
Formoterol	8.66	0.18	1.12	0.08	1.07	1.33	0.26	3
Clenbuterol	7.88	0.08	0.24	0.05	1.4	-0.11	0.08	6
Salbutamol	6.34	0.11	0.37	0.07	1.17	-1.43	0.02	3
Levosalbutamol	6.59	0.12	0.35	0.08	1.41	-1.26	0.23	3
Tulobuterol	6.76	0.17	0.05	0.01	1.44	-1.9	0.13	5
Isoetharine	6.68	0.06	0.72	0.11	1.34	-0.81	0.2	3

1.2 Supplementary Table 2, continued: Summary pharmacology data, plate-matched Δlog(Emax/EC50)

	1321N1: Functional desensitization							
	Mean pEC50	pEC50 (SD)	Mean Emax	Emax (SD)	Mean Hill slope	Mean Isoprenaline Δlog(Emax/EC50)	95% CI Isoprenaline Δlog(Emax/EC50)	n
Isoprenaline	8.37	0.3	1.00	0.00	1.29	0	0	8
Adrenaline	7.63	0.12	1.08	0.02	1.01	-0.58	0.13	3
Noradrenaline	5.82	0.15	1.00	0.14	1.26	-2.35	0.03	4
Formoterol	9.48	0.37	0.87	0.05	1.45	1.07	0.12	3
Clenbuterol	8.48	0.04	0.81	0.04	1.13	0.25	0.13	3
Salbutamol	7.15	0.04	0.90	0.01	1.04	-1.08	0.19	3
Levosalbutamol	7.45	0.01	0.85	0.07	0.91	-0.8	0.13	3
Tulobuterol	7.23	0.22	0.48	0.14	1.16	-1.2	0.42	3
Terbutaline	6.8	0.58	0.91	0.12	0.92	-1.47	0.35	4
Isoetharine	7.24	0.25	0.99	0.03	1.15	-0.92	0.22	5

1.3 Supplementary Table 3, comparison of plate-matched versus parallel-design calculations of confidence intervals corresponding to Δlog(E_{max}/EC_{50}) values. The left side of the table presents data as shown in Supplementary Table 2. The right side of the table is provided as an example of an alternate experimental design, representative of experimental conditions where technical matching of test and reference agonists is not possible. Log(E_{max}/EC_{50}) values were averaged across a subset of six experiments with all agonists tested in parallel. Δlog(E_{max}/EC_{50}) and confidence intervals were then determined by one-way ANOVA (Fisher's LSD).

	**CHO-K1 recombinant β₁-AR**					
	**Plate-matched, full dataset** Δlog(E_{max}/EC_{50}) calculated relative to isoprenaline in the same plate			**Parallel-design subset ANOVA** Δlog(E_{max}/EC_{50}) calculated after averaging log values across n=6 replicates		
	Mean Δlog(E_{max}/EC_{50})	95% CI Δlog(E_{max}/EC_{50})	n	Mean Δlog(E_{max}/EC_{50})	95% CI Δlog(E_{max}/EC_{50})	n
Isoprenaline	0	N/A	21	N/A	N/A	6
Adrenaline	-1.24	-1.35 to -1.13	12	-1.26	-1.53 to -0.99	6
Noradrenaline	-1.06	-1.15 to -0.97	18	-1.02	-1.29 to -0.75	6
Dobutamine	-1.67	-1.74 to -1.6	14	-1.67	-1.94 to -1.40	6
Formoterol	-0.87	-1.02 to -0.72	11	-1.07	-1.34 to -0.80	6
Clenbuterol	-1.86	-1.9 to -1.82	16	-1.82	-2.09 to -1.55	6
Salbutamol	-3.16	-3.25 to -3.07	11	-3.14	-3.41 to -2.87	6
Tulobuterol	-2.74	-2.86 to -2.62	10	-2.70	-2.97 to -2.42	6

	**CHO-K1 recombinant β₂-AR**					
	**Plate-matched** Δlog(E_{max}/EC_{50}) calculated relative to isoprenaline in the same plate			**Parallel-design subset ANOVA** Δlog(E_{max}/EC_{50}) calculated after averaging log values across n=6 replicates		
	Mean Δlog(E_{max}/EC_{50})	95% CI Δlog(E_{max}/EC_{50})	n	Mean Δlog(E_{max}/EC_{50})	95% CI Δlog(E_{max}/EC_{50})	n
Isoprenaline	0	N/A	22	N/A	N/A	6
Adrenaline	-0.36	-0.43 to -0.29	13	-0.29	-0.51 to -0.07	6
Noradrenaline	-2.30	-2.39 to -2.21	19	-2.21	-2.43 to -1.99	6
Dobutamine	-2.26	-2.34 to -2.18	15	-2.22	-2.44 to -2.00	6
Formoterol	1.17	1.02 to 1.32	11	1.01	0.80 to 1.23	6
Clenbuterol	0.03	-0.03 to 0.09	17	0.11	-0.11 to 0.32	6
Salbutamol	-1.37	-1.46 to -1.28	12	-1.30	-1.52 to -1.08	6
Tulobuterol	-1.28	-1.42 to -1.14	10	-1.18	-1.40 to -0.97	6

1.4 Supplementary Table 4, calculation of ΔΔlog(E_{max}/EC_{50}) values. Df = degrees of freedom.

Supplementary Figure 2										
	Δlog(E_{max}/EC_{50}) CHO-K1/$β_1$-AR			Δlog(E_{max}/EC_{50}) CHO-K1/$β_2$-AR			ΔΔlog E_{max}/EC_{50}) $β_1$-AR minus $β_2$-AR			
	Mean	SEM	n	Mean	SEM	n	Mean	SEM	Df	95% CI
Adrenaline	-1.27	0.10	6	-0.32	0.06	6	-0.95	0.12	10	-1.2 to -0.7
Noradrenaline	-1.03	0.05	6	-2.22	0.06	6	1.19	0.08	10	1.0 to 1.4
Dobutamine	-1.66	0.06	6	-2.22	0.05	6	0.55	0.08	10	0.4 to 0.7
Formoterol	-1.08	0.05	6	0.99	0.06	6	-2.07	0.08	10	-2.3 to -1.9
Clenbuterol	-1.83	0.04	6	0.10	0.04	6	-1.93	0.06	10	-2.1 to -1.8
Salbutamol	-3.15	0.06	6	-1.33	0.06	6	-1.82	0.09	10	-2.0 to -1.6
Tulobuterol	-2.68	0.04	6	-1.15	0.06	6	-1.53	0.08	10	-1.7 to -1.4
Figure 6D										
	Δlog(E_{max}/EC_{50}) Arrestin Recruitment			Δlog(E_{max}/EC_{50}) cAMP PathHunter®			ΔΔlog E_{max}/EC_{50}) Arrestin minus cAMP in PathHunter®			
	Mean	SEM	n	Mean	SEM	n	Mean	SEM	Df	95% CI
Adrenaline	-0.46	0.06	3	-0.26	0.03	6	-0.20	0.07	7	-0.4 to 0.0
Noradrenaline	-2.58	0.09	3	-2.56	0.04	5	-0.02	0.1	6	-0.2 to 0.2
Levosalbutamol	-1.26	0.12	3	-1.06	0.07	4	-0.20	0.14	5	-0.6 to 0.2
Formoterol	1.33	0.13	3	1.17	0.26	5	0.16	0.29	6	-0.5 to 0.9
Clenbuterol	-0.11	0.04	6	0.02	0.02	5	-0.13	0.04	9	-0.2 to -0.03
Salbutamol	-1.43	0.01	3	-1.43	0.02	5	0.00	0.02	6	-0.1 to 0.1
Tulobuterol	-1.90	0.06	5	-1.33	0.02	4	-0.57	0.06	7	-0.7 to -0.5
Isoetharine	-0.81	0.10	3	-1.07	0.11	4	0.26	0.15	5	-0.1 to 0.7
Figure 7B										
	Δlog(E_{max}/EC_{50}) Desensitization 1321N1			Δlog(E_{max}/EC_{50}) cAMP 1321N1			ΔΔlog E_{max}/EC_{50}) Desensitization minus cAMP in 1321N1			
	Mean	SEM	n	Mean	SEM	n	Mean	SEM	Df	95% CI
Adrenaline	-0.58	0.07	3	-0.25	0.14	7	-0.33	0.16	8	-0.7 to 0.07
Noradrenaline	-2.35	0.01	4	-2.45	0.20	8	0.10	0.2	10	-0.3 to 0.5
Levosalbutamol	-0.80	0.07	3	-1.09	0.07	5	0.29	0.1	6	0.09 to 0.5
Formoterol	1.07	0.06	3	1.44	0.09	5	-0.37	0.11	6	-0.7 to -0.07
Clenbuterol	0.25	0.06	3	0.02	0.06	22	0.23	0.84	23	-1.5 to 1.9
Salbutamol	-1.08	0.10	3	-1.24	0.10	9	0.16	0.14	10	-0.1 to 0.5
Tulobuterol	-1.20	0.21	3	-1.42	0.09	20	0.22	0.23	21	-0.3 to 0.7
Isoetharine	-0.92	0.11	5	-1.05	0.10	5	0.13	0.15	8	-0.2 to 0.4
Terbutaline	-1.47	0.18	4	-1.55	0.16	6	0.08	0.24	8	-0.5 to 0.7

1.5 Supplementary Figure 1

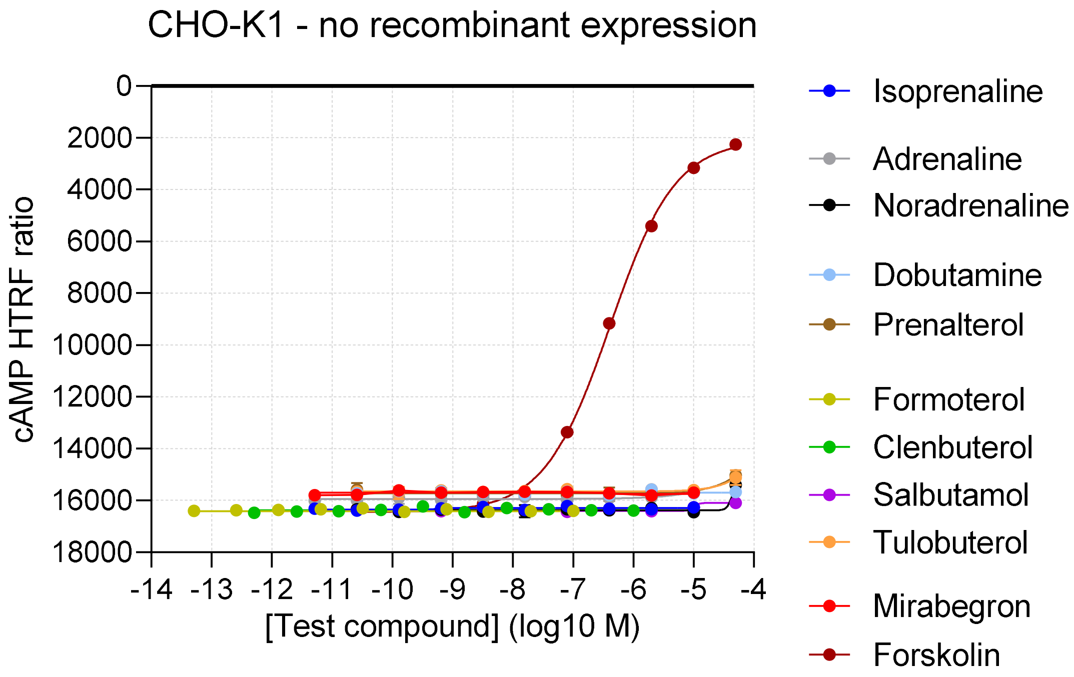

Supplementary Figure 1. CHO-K1 cells lacking recombinant receptor expression show no functional cAMP response to a panel of β-AR agonists.

1.6 Supplementary Figure 2

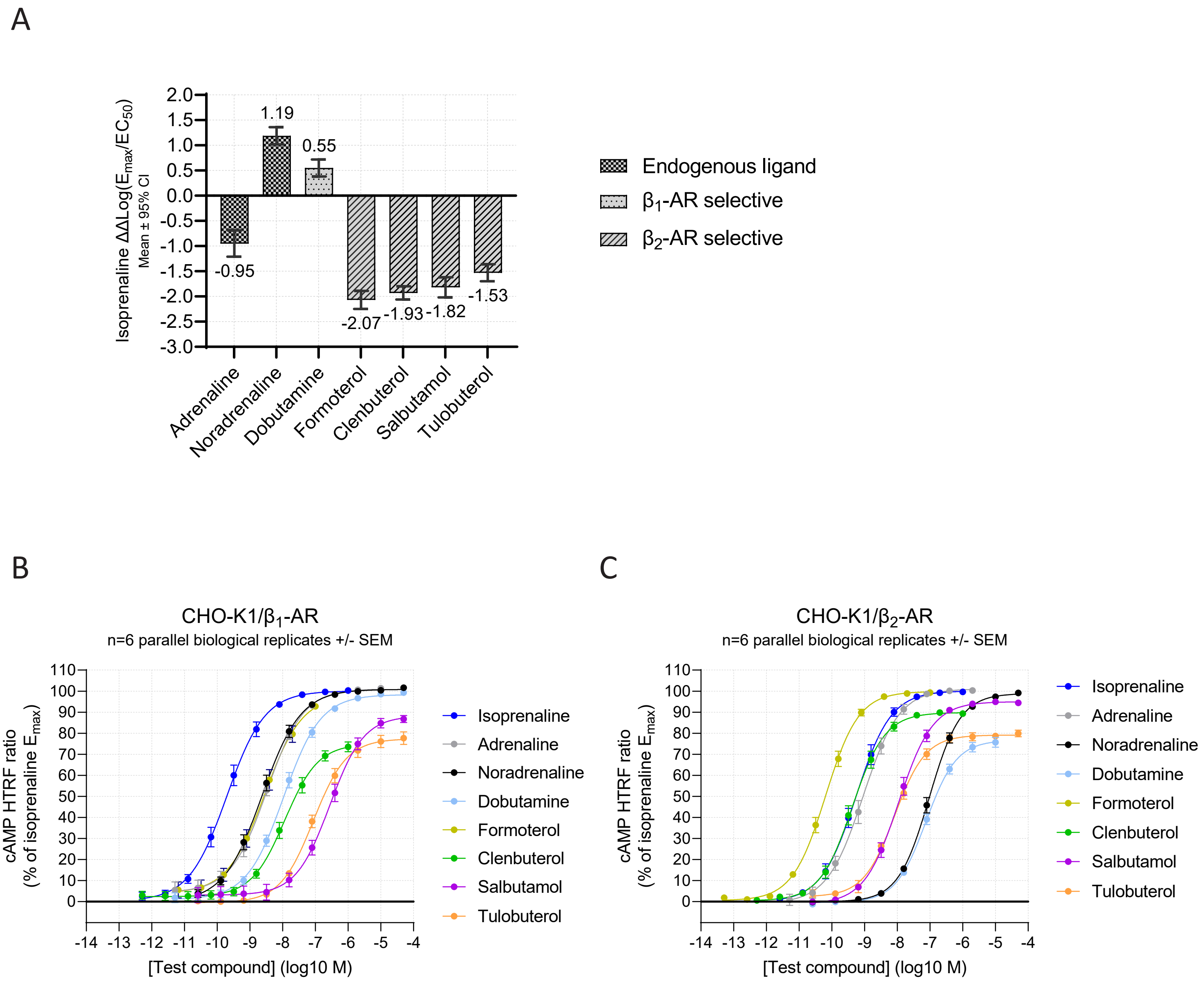

Supplementary Figure 2: (A) Analysis of ΔΔlog(Emax/EC50) values between receptors is a useful method to rank receptor selectivity across different expression systems. Displayed is a subset of data with agonists assayed in parallel to calculate ΔΔlog(Emax/EC50) by subtracting the mean values of Δlog(Emax/EC50) relative to isoprenaline at CHO-K1/β2-AR cells from that measured at CHO-K1/β1-AR cells. Resultant positive values indicate functional selectivity for β1-AR, and negative values indicate functional selectivity for β2-AR. Below are average concentration-response curves across the six experimental replicates. (B-C) Average concentration response curves: each data point represents mean ± SEM across six independent experiments in CHO-K1/ β1-AR and CHO-K1/β2-AR cells where the displayed agonists were tested in parallel.

1.7 Supplementary Figure 3

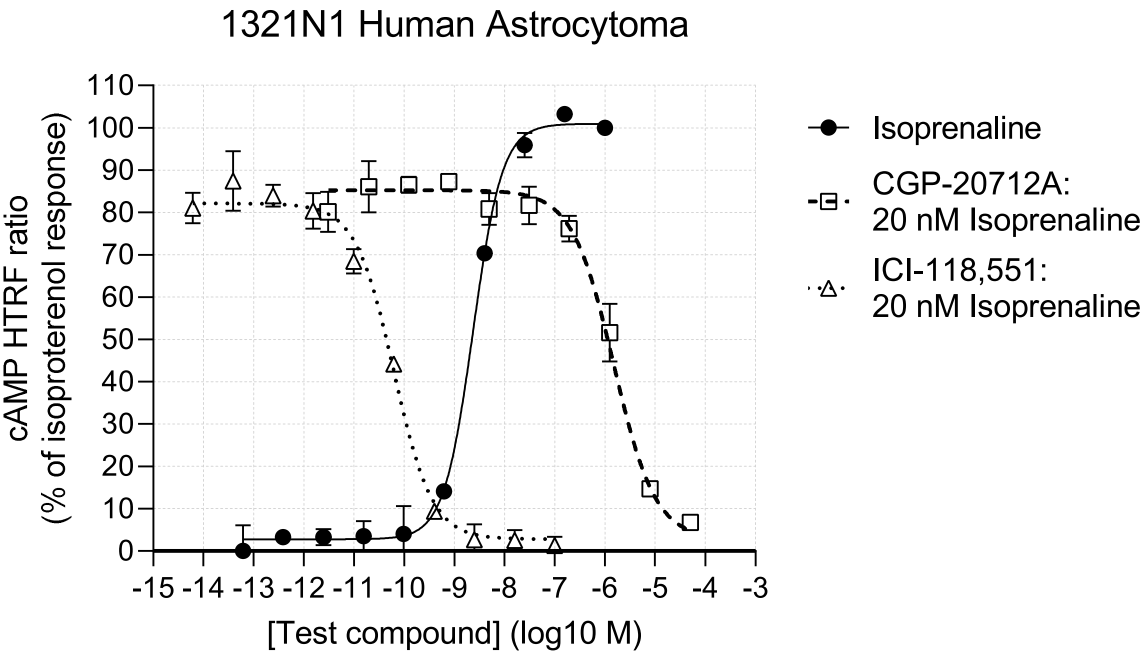

	IC_{50}	pKb
CGP-20712A	5.49 ± 0.29 (3.2 µM)	6.63 ± 0.1 (234 nM)
ICI-118,551	10.28 ± 0.12 (52 pM)	11.42 ± 0.24 (3.8 pM)

Supplementary Figure 3. Pre-incubation with the selective antagonists CGP-20712A (β_1-AR-selective) and ICI-118,551 (β_2-AR-selective) before cAMP stimulation with 20 nM isoprenaline (~EC_{80}) confirms that β_2-AR is highly expressed in 1321N1 cells.

1.8 Supplementary Figure 4

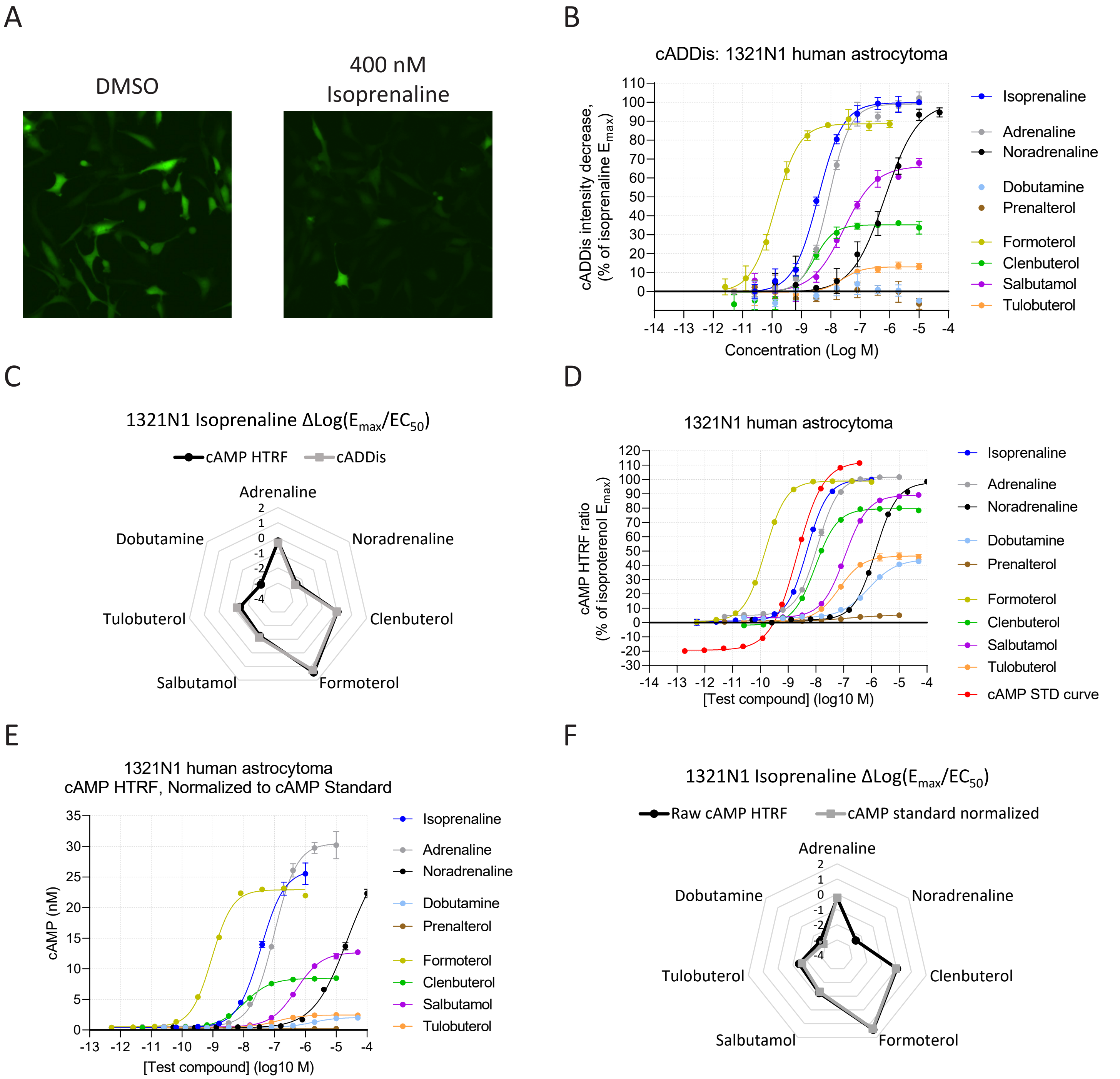

Supplementary Figure 4: (A) The cADDis biosensor (Montana Molecular) displays a decrease in fluorescence when bound to cAMP, induced by isoprenaline treatment of 1321N1 cells, which endogenously express β_2-AR. (B-C) Concentration-response curves to β-AR agonists in the cADDis assay were transformed to an agonist fingerprint that overlaid with cAMP measured by homogenous time-resolved fluorescence (HTRF). (D-E) Normalization of 1321N1-derived HTRF values to determine cAMP concentrations by interpolation from a cAMP standard curve (red). (F) Overlay of agonist fingerprints derived from raw and normalized HTRF values.

1.9 Supplementary Figure 5

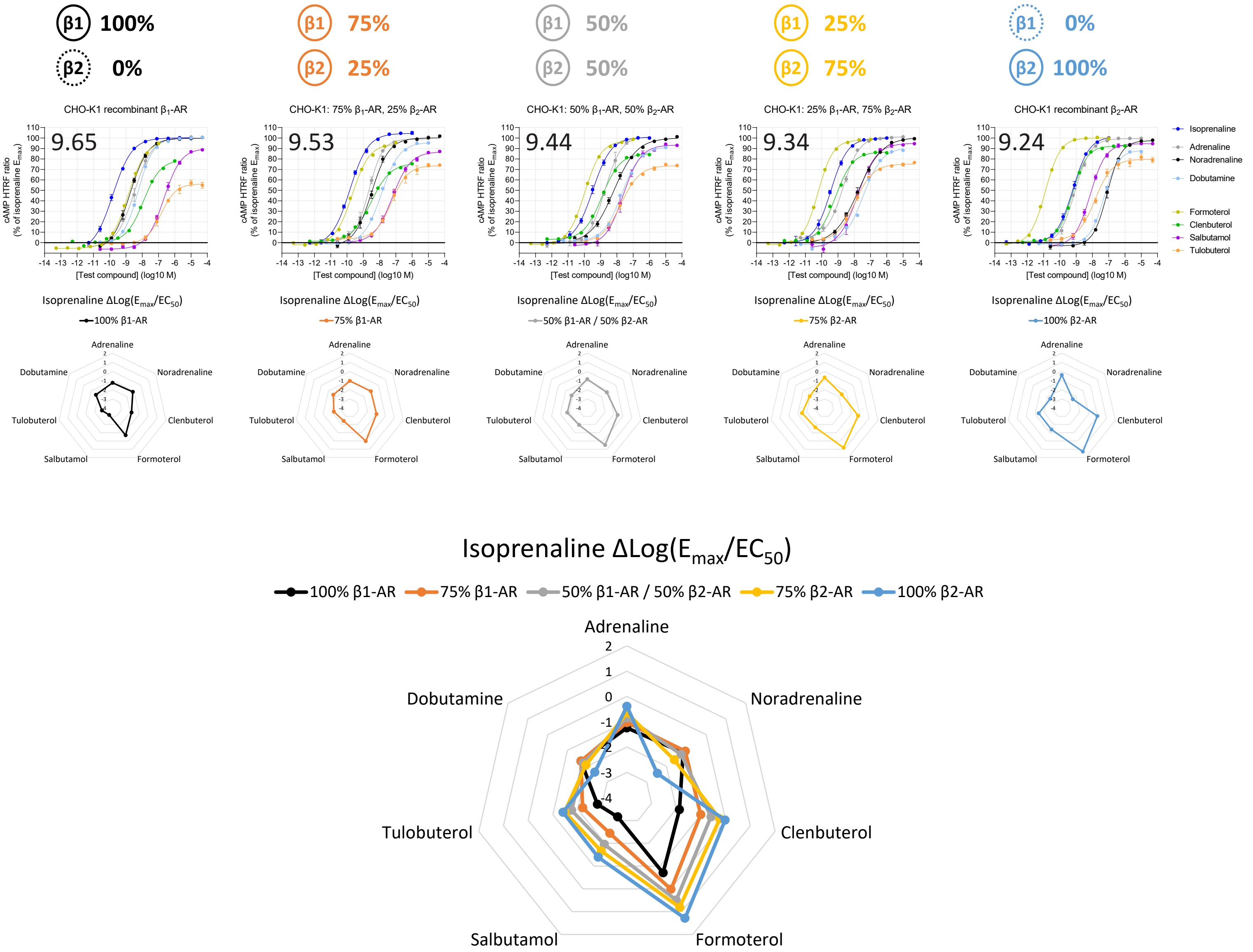

Supplementary Figure 5: Mixing populations of cells expressing either β_1-AR or β_2-AR (according to the percentages shown above each graph) results in intermediate values of isoprenaline pEC50 (graph insets) and $\Delta\log(E_{max}/EC_{50})$ for other agonists relative to isoprenaline. Thus, a mixed-population expression system gives an agonist fingerprint that appears as a hybrid of the two homogenous expression systems (lower panel).

1.10 Supplementary Figure 6

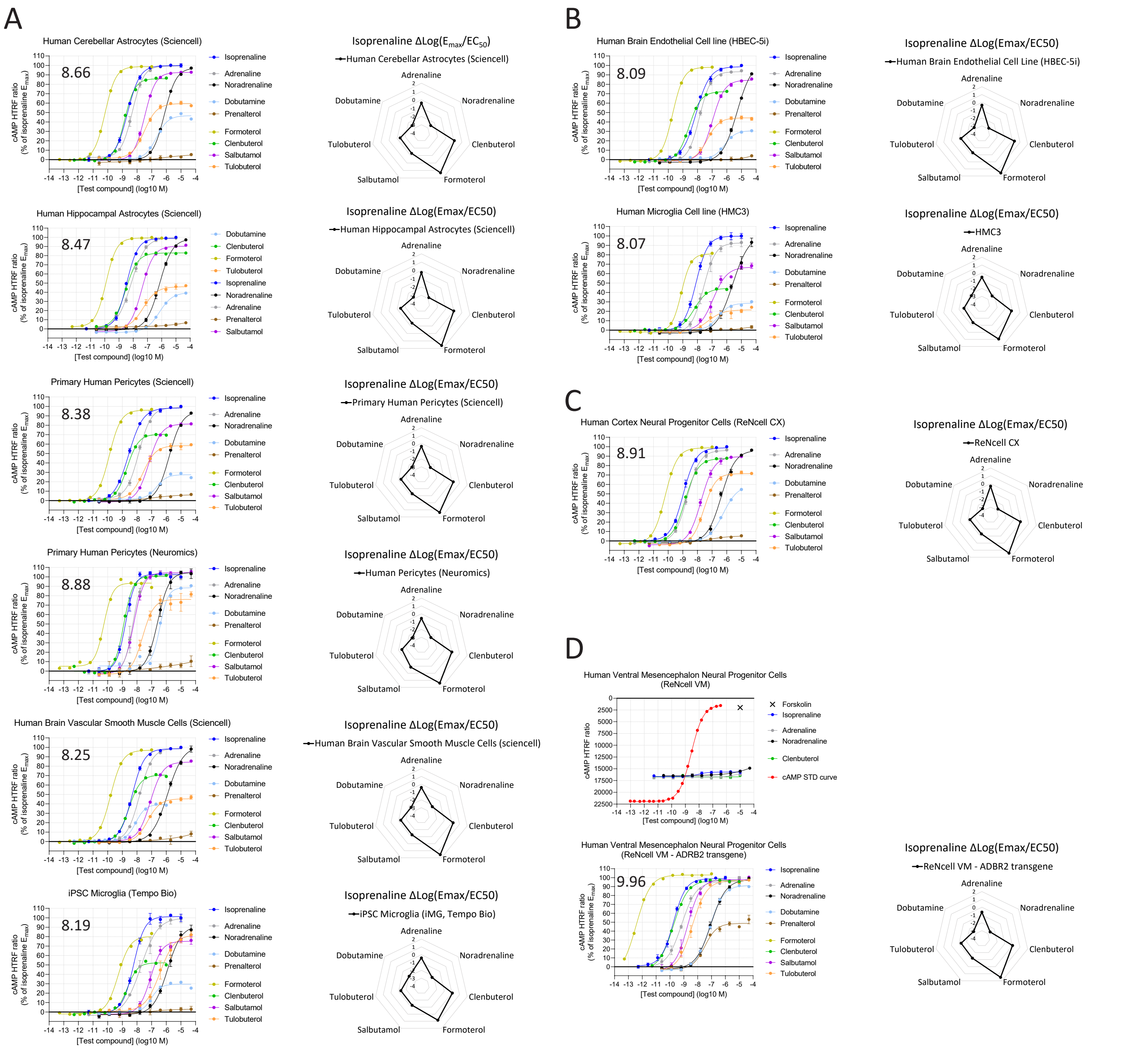

Supplementary Figure 6: Human brain-derived cells display predominantly β_2-AR activity. Displayed are concentration-response curves for a panel of β-AR agonists (graphs), their isoprenaline pEC50 values (insets) and corresponding $\Delta\log(E_{max}/EC_{50})$ agonist fingerprints (radar plots) tested in (A) human primary brain cells: hippocampal and cerebellar astrocytes, pericytes, vascular smooth muscle cells, and microglia; (B) cell lines derived from human brain tissue: HBEC-5i and HMC3, microvascular endothelial and microglial-derived cell lines, respectively; (C) neural progenitor cells derived from human cortex; or (D) neural progenitor cells derived from human ventral mesencephalon: before and after transgenic expression of ADRB2 (gene encoding β_2-AR).

1.11 Supplementary Figure 7

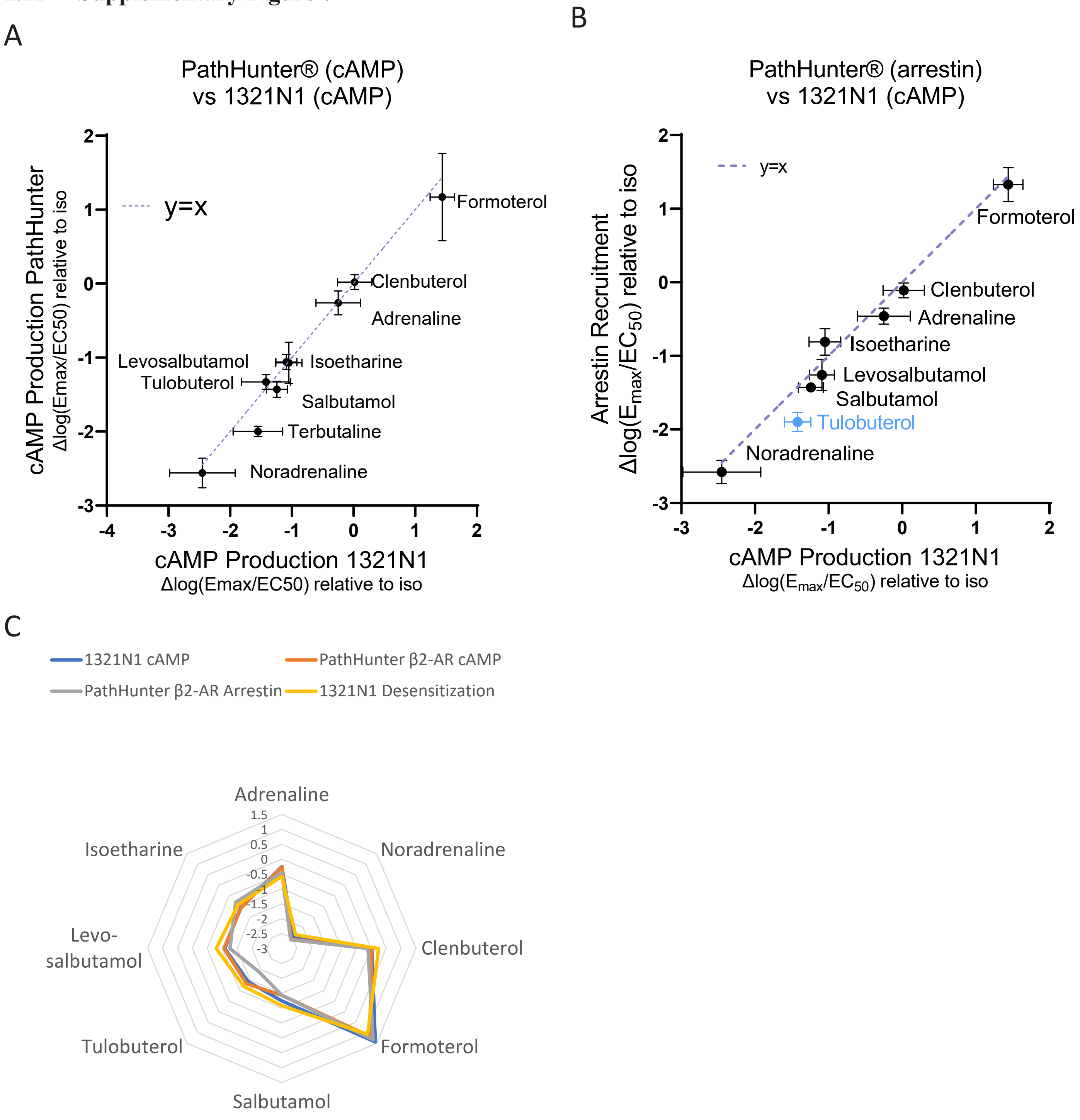

Supplementary Figure 7: (A) Correlation of $\Delta\Delta\log(E_{max}/EC_{50})$ values across a panel of β-AR agonists shows that cAMP production is coupled to β_2-AR similarly between 1321N1 and PathHunter® cell lines. (B) Similarly, arrestin recruitment in PathHunter® cell lines correlates with cAMP production in 1321N1 cells, with tulobuterol as an outlier due to low E_{max} and thus poor curve fits in the arrestin response. (C) Agonist fingerprints are similar across different β_2-AR-expressing cell types with endogenous or exogenous (fusion protein) receptors, and across signaling pathways (cAMP versus arrestin).
